# Unveiling of a cryptic *Dicranomyia (Idiopyga)* from northern Finland using integrative approach (Diptera, Limoniidae)

**DOI:** 10.3897/BDJ.2.e4238

**Published:** 2014-12-03

**Authors:** Jukka Salmela, Kari M Kaunisto, Varpu Vahtera

**Affiliations:** † Natural Heritage Services (Metsähallitus), Rovaniemi, Finland; ‡ Zoological Museum, Department of Biology, University of Turku, Turku, Finland

**Keywords:** Crane flies, DNA barcoding, Baltic coastal meadows, mires

## Abstract

The subgenus *Idiopyga* Savchenko, 1987 is a northern hemisphere group of short-palped crane flies (Diptera, Limoniidae). In the current article we describe a new species, *Dicranomyia (I.) boreobaltica* Salmela sp.n., and redescribe the male and female post-abdomen of a closely related species, *D. (I.) intricata* Alexander. A standard DNA barcoding fragment of 5′ region of the cytochrome *c* oxidase I (COI) gene of the new species is presented, whilst the K2P minimum distances between the new species and 10 other species of the subgenus were found to range from 5.1 to 15.7 % (mean 11.2 %). Phylogenetic analyses (parsimony and maximum likelihood) based on COI sequences support the identity of the new species and its close relationship with *D. (I.) intricata* and *D. (I.) esbeni* (Nielsen). The new species is known from the northern Baltic area of Finland. The new species has been mostly collected from Baltic coastal meadows but an additional relict population is known from a calcareous rich fen that was estimated to have been at sea level circa 600-700 years ago. *Dicranomyia (I.) intricata* (syn. *D. suecica* Nielsen) is a Holarctic species, occurring in the north boreal and subarctic vegetation zones in Fennoscandia.

## Introduction

The subgenus Idiopyga (Savchenko 1987) consists of 26 Holarctic (sub)species, of which 25 occur in the Palaearctic region (Oosterbroek 2014). Most of the species have wide ranges, such as *D. (I.) magnicauda* Lundström, *D. (I.) murina* (Zetterstedt) and *D. (I.) ponojensis* Lundström. A few species, or taxa that are recognised as subspecies, may have very restricted ranges (e.g. *D. (I.) melleicauda stenoptera* Savchenko [Savchenko 1970], *D. (I.) lulensis* (Tjeder) [Salmela 2012]) or disjunct ones (e.g. *D. (I.) esbeni* (Nielsen) [Salmela 2010, Devyatkov 2013]). *Dicranomyia (Idiopyga)* species are characterised by a complicated structure of male hypopygium, having appendages on their ventral gonostylus and gonocoxite. The female cerci are very short in species such as *D. (I.) intricata* Alexander and *D. (I.) lulensis* whereas a normal length (i.e. of normal length compared to other *Dicranomyia* species) in e.g. *D. (I.) halterella* Edwards and *D. (I.) ponojensis*. Species of the subgenus occur around temperate and subarctic wetlands (e.g. Alexander 1927, Tjeder 1958, Przhiboro 2003, Salmela 2011b), and some species favour or tolerate elevated concentration of salt (brackish water or salt marshes, Salmela 2010, Devyatkov 2013).

*Dicranomyia (I.) intricata* was reported from Finland by Nieminen (Nieminen 2008) in his M.Sc. thesis and the status of this species on the Finnish list of crane flies was later complemented and verified by Salmela (Salmela 2011a). However, during a project aiming to DNA barcode of (?) all Finnish crane fly species (http://www.finbol.org/eng/ENG_finbol.html), an abnormal intraspecific divergence was noted among the sequenced *D. (I.) intricata* specimens. Followed up by morphological examinations of voucher specimens, it was obvious that instead of one species there were two, distinguishable on both molecular and morphological basis (Salmela 2013). Examination of available type material (*D. (I.) intricata*, *D. suecica* (Nielsen)) allowed us to associate our morphospecies to taxonomic species. In the current article we describe a new Dicranomyia species from Finland and redescribe male and female post-abdomen of *D. (I.) intricata*. The new species is hitherto known from the northern Baltic area whereas *D. (I.) intricata* has a Holarctic range. To summarize, the approach we use is integrative (see e.g. Yeates et al. 2010). The description is based on integrative approach combining morphology, digital photographs, DNA barcodes and phylogenetic analysis.

## Materials and methods

The morphological terminology used here mainly follows Alexander and Byers 1981. Terminology of some special parts of male genitalia was taken from Devyatkov 2013 and female genitalia from Kotrba 2000, Stary 2004. The following acronyms for museums and collections are used: USNM – Smithsonian Institution, National Museum of Natural History, Washington DC, USA; ZMUC – Zoological Museum, University of Copenhagen, Copenhagen, Denmark; ZMUT – Zoological Museum, University of Turku, Turku, Finland; JES – Private collection of Jukka Salmela, Rovaniemi, Finland; JSO – Private collection of Jaroslav Starý, Olomouc, Czech Republic. All material deposited in ZMUT and JES are in 70% ethanol, other studied material was dry and pinned. Description is based on specimens preserved in ethanol; male and female hypopygia were macerated in KOH and are preserved in glycerol. Measurements of palpomeres, antennal segments and legs are based on a single specimen.

Layer photos were taken using an Olympus E520 digital camera, attached to an Olympus SZX16 stereomicroscope. Digital photos were captured using the programmes Deep Focus 3.1 and Quick PHOTO CAMERA 2.3. Layer photos were finally combined with the program Combine ZP.

A 658 bp fragment of mitochondrial protein-encoding cytochrome *c* oxidase subunit I (COI) was sequenced from a total of 22 *Dicranomyia* specimens and one *Metalimnobia* specimen. Legs or 2–3 abdominal segments of the specimens were placed in 96% ethanol in a 96-well lysis microplate and dispatched to the Canadian Centre for DNA Barcoding, Biodiversity Institute of Ontario where DNA was extracted and sequenced using standard protocols and primers (deWaard et al. 2008). The fragment was successfully amplified for all taxa except for *D. (I.) esbeni* for which the last 51 basepairs were missing due to sequencing problems. The new sequences are deposited in GenBank under accession numbers KP064165-KP064187 (Table 1) and are also available in Suppl. material 2.

### Phylogenetic analyses

*Parsimony approach*

Since the number of studied taxa was only 23, no heuristic methods were needed for the parsimony analysis. This allowed us to explore all possible evolutionary hypotheses for our data via explicit enumeration (branch and bound) analysis in TNT (Tree Analysis using New Technology) version 1.1 (Goloboff et al. 2008). Nodal support was measured by jackknife resampling (Farris et al. 1996) using 1000 replicates.

*Maximum likelihood approach*

Maximum likelihood analysis was conducted with RAxML ver. 8.0.22 (Stamatakis 2014) in the CIPRES Science Gateway (Miller et al. 2010). A unique general time-reversible (GTR) model of sequence evolution was specified and 100 independent searches were conducted. Nodal support was estimated via the rapid bootstrap algorithm (applying the Majority Rule Criterion) using the GTR-CAT model (Stamatakis et al. 2008).

## Taxon treatments

### Dicranomyia (Idiopyga) boreobaltica

Salmela sp. n.

urn:lsid:zoobank.org:act:5537A033-CC12-41C0-869C-F603D8775005

#### Materials

- **Type status:** Holotype. **Occurrence:** catalogNumber: JES-20120094; recordedBy: T. Nieminen; individualCount: 1; sex: male; **Taxon:** genus: Dicranomyia; subgenus: Idiopyga; specificEpithet: boreobaltica; scientificNameAuthorship: Salmela; **Location:** country: Finland; stateProvince: Ostrobothnia borealis pars ouluensis; verbatimLocality: Oulunsalo, Papinkari; verbatimLatitude: 64.9060; verbatimLongitude: 25.3764; verbatimCoordinateSystem: decimal degrees; verbatimSRS: WGS84; **Event:** samplingProtocol: Malaise trap; eventDate: 2005-8-11/10-8; habitat: Baltic coastal meadow; **Record Level:** institutionCode: ZMUT
- **Type status:** Paratype. **Occurrence:** recordedBy: T. Nieminen; individualCount: 1; sex: male; **Taxon:** genus: Dicranomyia; subgenus: Idiopyga; specificEpithet: boreobaltica; scientificNameAuthorship: Salmela; **Location:** country: Finland; stateProvince: Ostrobothnia borealis pars ouluensis; verbatimLocality: Hailuoto, Pökönnokka; verbatimLatitude: 65.0790; verbatimLongitude: 24.8883; verbatimCoordinateSystem: decimal degrees; verbatimSRS: WGS84; **Event:** samplingProtocol: Malaise trap; eventDate: 2005-8-11/10-8; habitat: Baltic coastal meadow; **Record Level:** institutionCode: JSO
- **Type status:** Paratype. **Occurrence:** recordedBy: T. Nieminen; individualCount: 1; sex: male; **Taxon:** genus: Dicranomyia; subgenus: Idiopyga; specificEpithet: boreobaltica; scientificNameAuthorship: Salmela; **Location:** country: Finland; stateProvince: Ostrobothnia borealis pars ouluensis; verbatimLocality: Hailuoto, Pökönnokka; verbatimLatitude: 65.0790; verbatimLongitude: 24.8883; verbatimCoordinateSystem: decimal degrees; verbatimSRS: WGS84; **Event:** samplingProtocol: Malaise trap; eventDate: 2005-8-11/10-8; habitat: Baltic coastal meadow; **Record Level:** institutionCode: ZMUT
- **Type status:** Paratype. **Occurrence:** catalogNumber: DIPT-JS-2014-0248; recordedBy: J. Salmela; individualCount: 1; sex: male; **Taxon:** genus: Dicranomyia; subgenus: Idiopyga; specificEpithet: boreobaltica; scientificNameAuthorship: Salmela; **Location:** country: Finland; stateProvince: Ostrobothnia borealis pars borealis; verbatimLocality: Tornio, Isonkummunjänkä Mire Conservation Area, Kusiaiskorpi; verbatimLatitude: 65.8880; verbatimLongitude: 24.4792; verbatimCoordinateSystem: decimal degrees; verbatimSRS: WGS84; **Event:** samplingProtocol: Malaise trap; eventDate: 2013-8-1/9-26; habitat: Rich fen, rusty spring; **Record Level:** institutionCode: JES
- **Type status:** Paratype. **Occurrence:** catalogNumber: DIPT-JS-2014-0251; recordedBy: J. Salmela; individualCount: 1; sex: female; **Taxon:** genus: Dicranomyia; subgenus: Idiopyga; specificEpithet: boreobaltica; scientificNameAuthorship: Salmela; **Location:** country: Finland; stateProvince: Ostrobothnia borealis pars borealis; verbatimLocality: Tornio, Isonkummunjänkä Mire Conservation Area, Kusiaiskorpi; verbatimLatitude: 65.8880; verbatimLongitude: 24.4792; verbatimCoordinateSystem: decimal degrees; verbatimSRS: WGS84; **Event:** samplingProtocol: Malaise trap; eventDate: 2013-8-1/9-26; habitat: Rich fen, rusty spring; **Record Level:** institutionCode: JES
- **Type status:** Paratype. **Occurrence:** recordedBy: J. Salmela; individualCount: 1; sex: female; **Taxon:** genus: Dicranomyia; subgenus: Idiopyga; specificEpithet: boreobaltica; scientificNameAuthorship: Salmela; **Location:** country: Finland; stateProvince: Ostrobothnia borealis pars borealis; verbatimLocality: Tornio, Isonkummunjänkä Mire Conservation Area, Kusiaiskorpi; verbatimLatitude: 65.8880; verbatimLongitude: 24.4792; verbatimCoordinateSystem: decimal degrees; verbatimSRS: WGS84; **Event:** samplingProtocol: Malaise trap; eventDate: 2013-8-1/9-26; habitat: Rich fen, rusty spring; **Record Level:** institutionCode: ZMUT
- **Type status:** Other material. **Occurrence:** catalogNumber: DIPT-JS-2014-0114; recordedBy: J. Salmela; individualCount: 7; sex: 4 females, 3 males; **Taxon:** genus: Dicranomyia; subgenus: Idiopyga; specificEpithet: boreobaltica; scientificNameAuthorship: Salmela; **Location:** country: Finland; stateProvince: Ostrobothnia borealis pars borealis; verbatimLocality: Tornio, Isonkummunjänkä Mire Conservation Area, Kusiaiskorpi; verbatimLatitude: 65.8880; verbatimLongitude: 24.4792; verbatimCoordinateSystem: decimal degrees; verbatimSRS: WGS84; **Event:** samplingProtocol: Malaise trap; eventDate: 2013-8-1/9-26; habitat: Rich fen, rusty spring; **Record Level:** institutionCode: JES

#### Description

*Dicranomyia (Idiopyga) intricata* Nieminen 2008: 24 (misidentification)

*Dicranomyia (Idiopyga) cf. intricata* Salmela 2013: 38 (preliminary annotation to the Finnish list)

**Figure 1.**
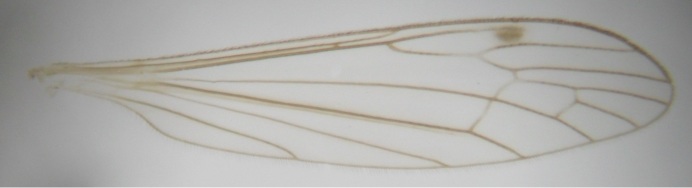
*Dicranomyia (I.) boreobaltica* Salmela sp.n., male wing, length 6.5 mm. DIPT-JS-2014-0114.

Male. **Head.** Vertex dark brown, with short black setae. Rostrum light brown with a few short dark setae. Palpus 5-segmented; first palpomere very short, globular, 1.5 times wider than long; other palpomeres elongated, p2 length 140 µm, p3 100 µm, p4 100 µm and p5 120 µm. First palpomere with a long ventral seta, approximately 2 times longer than width of palpomere. Second and third palpomeres with 5 setae, arranged in the apical half of segments. Fourth palpomere bearing ca. 12 setae and p5 with 13-15 setae, most of these on the apices of the segments. Antennae 14-segmented, dark brown, segments bearing black setae mostly exceeding width of respective segment; setae straight on scape (ca. 10 setae) and pecidel (ca. 15 setae), straight or curved on flagellomeres (ca. 5 setae on each flagellomere). Scape cylindrical, length 200 µm, width 75 µm, pedicel wider apically than basally, length 115 µm, width 75 µm. Flagellomeres oval, longer than wide; f1 length 120 µm, width 65 µm, f2 length 8 µm, width 5 µm, f10 length 110 µm, width 40 µm. **Thorax** mainly dark brown. Prescutum dark brown, only small yellowish spots on hind lateral corners. Scutum dark brown with longitudinal yellow median line and yellow lateral spots near wing base. Mediotergite and anepisternum dark brown, mediotergite sometimes with narrow yellowish anterior margin. Laterotergite and anepimeron yellowish brown. Katepisternum bicolored: anterior half dark brown, posterior half yellowish brown. Fore coxa brown, mid and hind coxae yellowish brown. Femorae light brown or brown, tibiae and tarsi dark brown. Length of fore femora 4500 µm, tibia 5250 µm, t1 3500 µm, t2 1100 µm, t3 875 µm, t4 300 µm, t5 175 µm, claw 130 µm. Length of mid femora 5575 µm, tibia 5625 µm, t1 3200 µm, t2 1150 µm, t3 625 µm, t4 275 µm, t5 175 µm, claw 130 µm. Length of hind femora 5600 µm, tibia 5750 µm, t1 3050 µm, t2 1150, t3 650 µm, t4 275 µm, t5 178 µm, claw 130 µm. Halter grayish-brown. Wing clear, veins light brown - brown, pterostigma brown (Fig. 1). Apical part of Sc1, R1, Rs, R3, R4+5, M1+2, M3, CuA1, CuA2, apices of A1 and A2 with macrotrichia, other veins bare. Sc1 ending in C before or opposite base of Rs (Fig. 1). Wing length 6.0-6.5 mm. **Abdomen** light brown - dark brown, tergites mainly dark, anterior sternites lighter than caudal sternites. Both sternites and tergites covered with short brown setae. 9th tergite and proctiger as in Fig. 2. Gonocoxite and gonostylus with complicated structure. Gonocoxite dark brown, sparsely covered with dark setae. Ventromesal lobe of gonocoxite consisting of two main branches, the main lobe (lgx) and its appendage (algx, Fig. 3). The main lobe (lgx) is rod-like, straight and elongated, apex angular, medially with a patch of hyaline curly setae. The appendage (algx) with two branches, proximal branch smaller, having a small hairy lobe, caudal branch larger in size, apically with tuft of rather long hyaline setae (Fig. 3a). Inner appendage of gonocoxite (iagx, Fig. 4a, b, c) sclerotized, curved structure, apically with number of stout, short setae; apex of iagx rounded and slightly wider than its stalk. Gonostylus consisting of two main lobes (dorsal [dg] and ventral [vg]), ventrobasal lobe of ventral gonostylus (lvg), rostral prolongation (rostrum) of ventral gonostylus (rm) and subrostral prolongation of ventral gonostylus (srm) (Figs 3b, 4). Dg long, narrow, pointed and bare, vg ball-like, weakly sclerotized, bearing setae and microtrichia (Figs 3b, 4a). Lvg tail-like, sinuous and weakly sclerotized, having patches of hyaline setae both basally and apically; apex of lvg oval (Fig. 3). Basal part of rm dorsally covered with dark brown plate, rm light brown, well sclerotized and elongated, bearing two strong spines; apex of rm rounded and rather narrow, bearing a few short setae (Fig. 4a). Srm strongly sclerotized, approximately as long as rm, projected proximad (i.e. toward parameres), being widest medially; srm with number of median and subapical stout black spines; apex rounded in dorsal view, bearing one black and two hyaline stout setae (Fig. 4c, d). Ventral surface of aedeagus bearing hyaline setosity, lateral margin of parameres weakly serrated (Fig. 5).

Female. In general, similar to male. Wing length 6.5 mm. Cerci short, ca. 240 µm in length. Infra-anal plate with a strong caudal peak (Fig. 6a). Other parts of the female post-abdomen are presented in Fig. 6.

#### Diagnosis

Brownish, small species, very close to *D. (I.) intricata*. Ventrobasal lobe of ventral gonostylus sinuous, apex oval. Inner appendage of gonocoxite apically rounded. Rostral prolongantion of ventral apically rather narrow and subrostral prolongation simple, not bilobed, bearing dark stout spines. Female infra-anal plate with strong caudal peak.

#### Etymology

Boreo (*borealis*, Latin)= north, baltica (Latin)= referring to the Baltic Sea. The species is so far known from the northern Baltic area. The species name is deemed to be a latinized adjective in nominative singular.

#### Distribution

**Figure 14.**
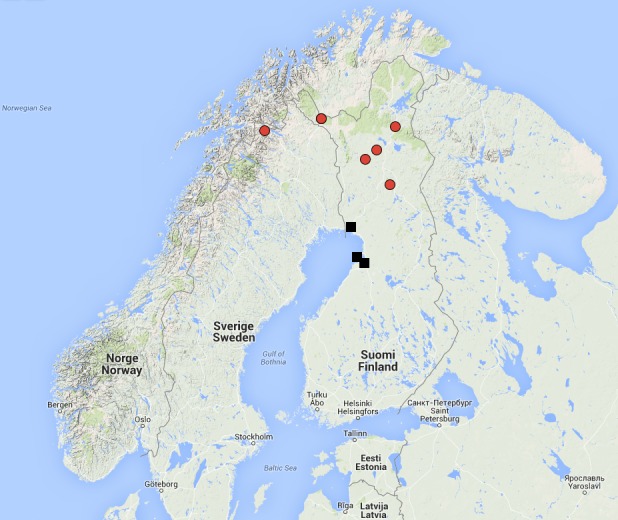
Collecting sites of *Dicranomyia (I.) boreobaltica* Salmela sp.n. (black squares) and *D. (I.) intricata* Alexander (red dots) in Fennoscandia. The map was drawn by using Google Maps.

European, only known from Finland. The species is hitherto known from five separate localities; four of these are shore meadows in Oulunsalo and Hailuoto island (see Nieminen 2008), and the fifth locality is in Tornio, a rich fen ca. 12 km inland from the coast line, 15 m above sea level (Fig. 14).

#### Ecology

The species is probably halophilous, occurring in Baltic coastal meadows characterised by vascular plants such as *Phragmites australis*, *Lysimachia thyrsiflora*, *Eleocharis palustris*, *Carex halophila* and *C. paleacea* (Nieminen 2008, as *D. (I.) intricata*). These coastal meadows are produced by a phenomenon called land uplift, that is, the rebound of earth's crust after the retreating of the ice sheet; in the Bothnian Bay the rate of land uplift is about 8 mm/year (Rehell 2006). In addition to the meadows influenced by brackish water, the species has been collected from a calcareous rich spring fen. This rich spring fen is known to have high concentrations of e.g. Ca (53 mg/l), Na (5.3 mg/l), Fe (32 mg/l) and having high specific conductivity (42.7 mS/m), alkalinity (4.85 mmol/l) and pH (7.9, T. Sallantaus, personal communication). This spring fen is located quite close to the current shore line, and extrapolating from Okkonen 2003 (fig. 21), one may estimate that this fen was on the Baltic shore some 600-700 years ago. It may be that high concentrations of dissolved minerals in the fen resemble brackish water habitat, allowing the survival of this halophilous crane fly species. It may thus be assumed that *D. (I.) boreobaltica* Salmela sp.n. is a recent relict species in the fen. It should be noted that some plants typical for the Baltic shores or brackish water have isolated populations on calcareous ponds or mires far from coastal areas (e.g. *Tricloghin maritima*, *Potamogeton filiformis*, Hämet-Ahti et al. 1998)

#### Conservation

Due to its apparent rarity, that is, small area of occupancy and extent of occurrence, the species could most likely be assessed as a threatened species according to IUCN criteria. Habitats of this species are highly endangered, usually small and isolated. There are a total of ca. 4200 ha of Baltic coastal meadows along the Finnish coast, and all such habitat types are red-listed (Schulman et al. 2008). Also spring fens are threatened habitats (Leka et al. 2008). Furthermore, Salmela (Salmela 2005) studied adult crane fly fauna of 20 springs, of which 10 were calcareous springs, only some 30–60 km northeast from Kusiaskorpi rich fen, and *D. (I.) boreobaltica* Salmela sp.n. was absent from the samples. This and other negative records (i.e. absence) from >500 Malaise trapping sites in Finnish wetlands (Salmela 2012, Salmela 2013, J. Salmela unpublished) indicate a very restricted range of this species. In a matter of fact, there are some endemic or highly disjunct plant (e.g. *Alisma wahlenbergii*, *Euphrasia bottnica*, *Primula nutans*) and insect (*Elachista vonschantzi*, *Holopyga metallica*, *Macroplea pubipennis*) species in the Baltic coastal areas (Hultén 1950, Dahl 1998, Mutanen 2003, Kölsch et al. 2006, Paukkunen et al. 2014). Hence, by using the above mentioned plants and insects as surrogates, *D. (I.) boreobaltica* Salmela sp.n. could either be i) a recently evolved allopatric species that survived Pleistocene glaciations and is currently only present in the Baltic area or ii) a disjunct species having populations in other (coastal) areas.

#### Taxon discussion

Based on morphology and COI sequence divergence, the new species is very closely related to the Holarctic species *D. (I.) intricata*. As already stated in the title of this article, the new species is cryptic, meaning that it is hard to distinguish from its sister species by morphological characters. Strictly speaking, cryptic species may mean taxa that are morphologically indisguishable (Pfenninger and Schwenk 2007, but see Tan et al. 2010), but the new species described here can be separated from its sister species by using a genetic marker (barcoding region of COI) and morphology. However, morphological differences between these two species are not great and the only reliable diagnostic characters are found from male and female genitalia. These two species are allopatric, their closest known populations lay some 180 km apart. Despite these species not being from sympatric populations, we assume that their differences are well sufficient to keep their gene pools separated even in the case of possible secondary contact. Their COI divergence or K2P distance (5 %) is far too high to be considered as an intraspecific variation among majority of other insects (e.g. Hausmann et al. 2011, Park et al. 2011) or crane flies (Pilipenko et al. 2012). Instead, intraspecific variation among insects is typically smaller than 2 % and higher COI divergence usually indicates two separate species (Mutanen et al. 2013, Pentinsaari et al. 2014). Considering morphology, there is a recent case study from Israel (Stary et al. 2012) showing that two closely related, allopatric *Phyllolabis* crane flies were treated as separate species although they have almost identical genitalia and the closest populations of these species live only 30 km apart. In Israel, the species were separated by a dispersal barrier (Rift Valley, Stary et al. 2012); in Fennoscandia, *D. (I.) boreobaltica* Salmela sp.n. and *D. (I.) intricata* are not separated by a distinct barrier, they have non-overlapping ranges perhaps because of biogeographic factors driven by climate (see e.g. Luoto et al. 2006, Väisänen et al. 1992) and availability of habitat (brackish water, calcareous springs in the vicinity of coast line).

External characters, such as wing venation and body coloration, between *D. (I.) boreobaltica* Salmela sp.n. and *D. (I.) intricata* are practically identical. The most important differences in male and female post-abdomen between the species are summarized in Table 2. Among other *D. (Idiopyga)* species, the new species is quite close to *D. (I.) esbeni*. Besides other details, the ventrobasal lobe of gonocoxite in *D. (I.) esbeni* is sinuous (see Stary 2007, fig. 1), and rather straight in *D. (I.) boreobaltica* Salmela sp.n. Dicranomyia (I.) melleicauda complicata de Meijere is also quite close to the new species, but has rather stout iagx and apically wide lvg (see de Meijere 1919, plates 5-6). Males of other species are easily separated from the new species based on differences in the stucture of hypopygium. Considering females, we refrain from further discussion due to the lack of comparative material.

#### DNA barcode

Standard 5′ region (658 bp) of the cytochrome *c* oxidase I (COI) sequence of *Dicranomyia (I.) boreobaltica* Salmela sp.n. BOLD Sample ID JES-20120094, holotype specimen:

TACCTTATACTTTATTTTTGGAGCTTGAGCAGGAATAGTGGGAACTTCATTAAGTATTATTATTCGAGCAGAATTAGGACACCCAGGTGCATTAATTGGAGACGACCAGATTTATAATGTGGTAGTTACTGCCCATGCTTTTATTATAATTTTCTTTATAGTTATACCAATTATAATTGGAGGATTCGGTAATTGATTAGTTCCTTTAATATTAGGAGCCCCAGATATAGCTTTCCCTCGAATAAATAATATAAGTTTTTGAATACTTCCCCCTTCTTTAACTTTATTATTAGCTAGAAGCATAGTTGAAAACGGGGCAGGAACTGGCTGAACAGTATACCCTCCCCTTTCTTCTGGAATTGCCCATTCAGGGGCTTCTGTAGATTTAGCTATTTTTTCTCTTCACCTAGCAGGTATTTCTTCTATTTTAGGAGCTGTTAATTTTATTACAACTGTTATTAATATACGTTCAGCAGGAATTTCATTTGATCGAATACCATTATTTGTTTGATCAGTAGTAATTACTGCTATTTTATTGCTTTTATCACTTCCTGTTTTAGCCGGAGCTATTACAATATTATTAACAGATCGAAACTTAAATACTTCATTTTTTGATCCCGCAGGTGGAGGAGACCCTATTTTATATCAGCATTTATTT

Based on K2P (Kimura 1980) distances, the new species is closest to *D. (I.) intricata* (K2P distance 5.13 %), *D. (I.) esbeni* (7.16 %) and *D. (I.) magnicauda* (9.51 %); other distances within examined *D. (Idiopyga)* species range between 10.76 and 15.70 %.

### Dicranomyia (Idiopyga) intricata

Alexander, 1927

#### Materials

- **Type status:** Holotype. **Occurrence:** recordedBy: O. Bryant; individualCount: 1; sex: male; **Taxon:** genus: Dicranomyia; subgenus: Idiopyga; specificEpithet: intricata; scientificNameAuthorship: Alexander; **Location:** country: Canada; stateProvince: Alberta; verbatimLocality: Lesser Slave Lake; verbatimLatitude: 55.35; verbatimLongitude: -115.09; verbatimCoordinateSystem: decimal degrees; verbatimSRS: WGS84; **Event:** eventDate: 1924-8-1; **Record Level:** institutionCode: USNM
- **Type status:** Paratype. **Occurrence:** recordedBy: O. Bryant; individualCount: 1; sex: male; **Taxon:** genus: Dicranomyia; subgenus: Idiopyga; specificEpithet: intricata; scientificNameAuthorship: Alexander; **Location:** country: Canada; stateProvince: Alberta; verbatimLocality: Lesser Slave Lake, Grizzly mt.; minimumElevationInMeters: 914; **Event:** eventDate: 1924-8-15; **Record Level:** institutionCode: USNM
- **Type status:** Holotype. **Occurrence:** catalogNumber: 855; recordedBy: H. Frantz; individualCount: 1; sex: male; **Taxon:** genus: Dicranomyia; subgenus: Idiopyga; specificEpithet: suecica; scientificNameAuthorship: Nielsen; **Location:** country: Sweden; stateProvince: Abisko; verbatimLatitude: 68.35; verbatimLongitude: 18.79; verbatimCoordinateSystem: decimal degrees; verbatimSRS: WGS84; **Event:** eventDate: unknown; **Record Level:** institutionCode: ZMUC
- **Type status:** Other material. **Occurrence:** recordedBy: P.T. Bruggemann; individualCount: 1; sex: male; **Taxon:** genus: Dicranomyia; subgenus: Idiopyga; specificEpithet: intricata; scientificNameAuthorship: Alexander; **Location:** country: Canada; stateProvince: Yukon; verbatimLocality: Dawson; minimumElevationInMeters: 335; **Event:** eventDate: 1949-8-6; **Record Level:** institutionCode: USNM
- **Type status:** Other material. **Occurrence:** recordedBy: W.W. Moss; individualCount: 1; sex: male; **Taxon:** genus: Dicranomyia; subgenus: Idiopyga; specificEpithet: intricata; scientificNameAuthorship: Alexander; **Location:** country: Canada; stateProvince: British Columbia; verbatimLocality: Telegraph Creek; minimumElevationInMeters: 335; **Event:** eventDate: 1960-8-28; **Record Level:** institutionCode: USNM
- **Type status:** Other material. **Occurrence:** recordedBy: W.W. Moss; individualCount: 1; sex: male; **Taxon:** genus: Dicranomyia; subgenus: Idiopyga; specificEpithet: intricata; scientificNameAuthorship: Alexander; **Location:** country: Canada; stateProvince: British Columbia; verbatimLocality: Telegraph Creek, Sawmill Lake; **Event:** eventDate: 1960-8-18; **Record Level:** institutionCode: USNM
- **Type status:** Other material. **Occurrence:** recordedBy: O. Bryant; individualCount: 1; sex: male; **Taxon:** genus: Dicranomyia; subgenus: Idiopyga; specificEpithet: intricata; scientificNameAuthorship: Alexander; **Location:** country: Canada; stateProvince: Northwest Territories; verbatimLocality: Aklavik; **Event:** eventDate: 1931-8-27; **Record Level:** institutionCode: USNM
- **Type status:** Other material. **Occurrence:** catalogNumber: JES-20120082; recordedBy: J. Salmela; individualCount: 1; sex: female; **Taxon:** genus: Dicranomyia; subgenus: Idiopyga; specificEpithet: intricata; scientificNameAuthorship: Alexander; **Location:** country: Finland; stateProvince: Lapponia kemensis pars occidentalis; verbatimLocality: Kittilä, Mustaoja-Nunaravuoma Mire Conservation Area, Mustaoja W; verbatimLatitude: 67.6390; verbatimLongitude: 25.4277; verbatimCoordinateSystem: decimal degrees; verbatimSRS: WGS84; **Event:** samplingProtocol: sweep net; eventDate: 2009-8-19; habitat: rich flark fen; **Record Level:** institutionCode: ZMUT
- **Type status:** Other material. **Occurrence:** catalogNumber: DIPT-JS-2014-0336; recordedBy: J. Salmela; individualCount: 32; sex: 29 male, 3 female; **Taxon:** genus: Dicranomyia; subgenus: Idiopyga; specificEpithet: intricata; scientificNameAuthorship: Alexander; **Location:** country: Finland; stateProvince: Lapponia enontekiensis; verbatimLocality: Enontekiö, Tarvantovaara Wilderness Area, Tomuttirova W; verbatimLatitude: 68.6369; verbatimLongitude: 22.5381; verbatimCoordinateSystem: decimal degrees; verbatimSRS: WGS84; **Event:** samplingProtocol: sweep net; eventDate: 2009-8-26; habitat: swampy flark fen; **Record Level:** institutionCode: JES
- **Type status:** Other material. **Occurrence:** catalogNumber: JES-20110082; recordedBy: J. Salmela; individualCount: 1; sex: male; **Taxon:** genus: Dicranomyia; subgenus: Idiopyga; specificEpithet: intricata; scientificNameAuthorship: Alexander; **Location:** country: Finland; stateProvince: Lapponia enontekiensis; verbatimLocality: Enontekiö, Tarvantovaara Wilderness Area, Tomuttirova W; verbatimLatitude: 68.6369; verbatimLongitude: 22.5381; verbatimCoordinateSystem: decimal degrees; verbatimSRS: WGS84; **Event:** samplingProtocol: sweep net; eventDate: 2009-8-26; habitat: swampy flark fen; **Record Level:** institutionCode: ZMUT
- **Type status:** Other material. **Occurrence:** catalogNumber: DIPT-JS-2014-0337; recordedBy: J. Salmela; individualCount: 10; sex: 5 male, 5 female; **Taxon:** genus: Dicranomyia; subgenus: Idiopyga; specificEpithet: intricata; scientificNameAuthorship: Alexander; **Location:** country: Finland; stateProvince: Lapponia enontekiensis; verbatimLocality: Enontekiö, Tarvantovaara Wilderness Area, Tomuttirova N; verbatimLatitude: 68.6391; verbatimLongitude: 22.5518; verbatimCoordinateSystem: decimal degrees; verbatimSRS: WGS84; **Event:** samplingProtocol: sweep net; eventDate: 2009-8-26; habitat: intermediate rich flark fen; **Record Level:** institutionCode: JES
- **Type status:** Other material. **Occurrence:** catalogNumber: DIPT-JS-2014-0182; recordedBy: J. Salmela; individualCount: 1; sex: male; **Taxon:** genus: Dicranomyia; subgenus: Idiopyga; specificEpithet: intricata; scientificNameAuthorship: Alexander; **Location:** country: Finland; stateProvince: Lapponia kemensis pars orientalis; verbatimLocality: Sodankylä, Pomokaira-Tenniöaapa Mire Conservation Area, Syväkuru; verbatimLatitude: 67.8718; verbatimLongitude: 26.2126; verbatimCoordinateSystem: decimal degrees; verbatimSRS: WGS84; **Event:** samplingProtocol: Malaise trap; eventDate: 2013-8-15/9-19; habitat: spring fen; **Record Level:** institutionCode: JES
- **Type status:** Other material. **Occurrence:** catalogNumber: DIPT-JS-2014-0338; recordedBy: J. Salmela; individualCount: 4; sex: 1 male, 3 female; **Taxon:** genus: Dicranomyia; subgenus: Idiopyga; specificEpithet: intricata; scientificNameAuthorship: Alexander; **Location:** country: Finland; stateProvince: Ostrobothnia borealis pars borealis; verbatimLocality: Kemijärvi, Salmiaavanhete; verbatimLatitude: 66.9929; verbatimLongitude: 27.0578; verbatimCoordinateSystem: decimal degrees; verbatimSRS: WGS84; **Event:** samplingProtocol: sweep net; eventDate: 2009-8-15; habitat: rich flark fen; **Record Level:** institutionCode: JES
- **Type status:** Other material. **Occurrence:** catalogNumber: DIPT-JS-2014-0340; recordedBy: J. Salmela; individualCount: 1; sex: 1 female; **Taxon:** genus: Dicranomyia; subgenus: Idiopyga; specificEpithet: intricata; scientificNameAuthorship: Alexander; **Location:** country: Finland; stateProvince: Lapponia inariensis; verbatimLocality: Inari, Kaunispää; verbatimLatitude: 68.4461; verbatimLongitude: 27.4351; verbatimCoordinateSystem: decimal degrees; verbatimSRS: WGS84; **Event:** samplingProtocol: sweep net; eventDate: 2013-8-16; habitat: alpine wetland; **Record Level:** institutionCode: JES

#### Description

*Dicranomyia intricata* Alexander 1927: 221 (original description)

*Limonia (Dicranomyia) suecica* Nielsen 1953: 34 (original description)

*Limonia (Dicranomyia) suecica* Tjeder 1958: 160 (distribution, figure of hypopygium on p. 157)

*Dicranomyia (Idiopyga) intricata* Salmela 2011b: 224 (distribution, ecology)

The holotype specimen of *D. (I.) intricata* (Fig. 7a) is in good condition, dry and pinned, hypopygium is not detached. Alexander (Alexander 1927, fig. 1) permanently slide-mounted and illustrated his paratype specimen (Fig. 7b). The holotype of *Dicranomyia suecica* Nielsen was re-described and well illustrated by Tjeder (Tjeder 1958) and the species was proposed as a synonym of *D. (I.) intricata* by Savchenko et al. 1992. Unfortunately the male hypopogiym of *D. suecica* is lost (T. Pape, personal communication), but based on Tjeder's detailed illustrations this nomenclature can be verified. Alexander's original description is good, and there is no need to thoroughly re-describe this species. However, male and female genitalia are illustrated here and diagnostic characters are discussed under *D. (I.) boreobaltica* Salmela sp.n.

Male hypopygium. 9th tergite and proctiger as in Fig. 8. Gonocoxite dark brown, sparsely covered with dark setae. Ventromesal lobe of gonocoxite as in Fig. 9. The main lobe (lgx) club-like, straight and elongated, apex beak-like, having medially patch of hyaline curly setae (Fig. 9). The appendage of ventromesal lobe (algx) as in Fig. 9. Inner appendage of gonocoxite (iagx, Fig. 10a, b, c) sclerotized, curved, apically with a number of stout, short setae; apex of iagx bilobed. Structure of gonostylus as in *D. (I.) boreobaltica* Salmela sp.n., see Fig. 10a, b, c. Ventrobasal lobe of ventral gonostyle (lvg) tail-like, slightly sinuous, weakly sclerotized, having patches of hyaline setae both basally and apically; its stalk narrowing toward apex; apex of lvg spherical (Fig. 9). Rostrum (rm) light brown, apically widest, bearing two strong spines (Fig. 10a). Subrostral prolongation of ventral gonostyle (srm) strongly sclerotized, bilobed, very robust, approximately as long as rm, almost parallel with rm; srm with number of median and subapical stout black spines, apical spines are hyaline/light brown (Fig. 10b, c, d). Ventral surface of aedeagus bearing hyaline setosity, lateral margins of parameres rather strongly serrated (Fig. 11). See also Suppl. material 1.

Female postabdomen. Cerci and hypogynial valves, see Fig. 12.

#### Distribution

Holarctic. Known from Canada (Alberta, Northwest territories, British Columbia), Sweden (North Sweden, Abisko, Nielsen 1953, Tjeder 1958) and Finland. In Finland *D. (I.) intricata* is known from the north boreal ecoregion, both from the zone of coniferous forests and from the subarctic fell area in the northernmost part of the country (Fig. 14

#### Ecology

The original description of *D. (I.) intricata* (Alexander 1927) was based on material collected from "Muskeg" bogs. Muskegs are nutrient poor peatlands dominated by *Sphagnum* mosses (http://en.wikipedia.org/wiki/Muskeg). Most of the Finnish sampling sites are aapamires, that is, minerotrophic fens with wet, usually moss covered, flarks (hollows) and drier hummock-level strings. Most of the sites are intermediate rich or rich fens, characterised by brown mosses (e.g. *Warnstorffia*, *Scorpidium*, *Paludella*). The species was especially abundant on two closely lying intermediate rich, *Sphagnum* dominated aapamires in Enontekiö, NW Finnish Lapland, but single specimens were also caught along a spring and a headwater stream (Salmela 2011a). The species is on the wing from mid August to early September.

#### Conservation

*Dicranomyia (I.) intricata* is red-listed in Finland (NT, Penttinen et al. 2010). At the time of the assessment, it was not known that *D. (I.) intricata* is absent from the northern Baltic coastal area and is replaced there by a sibling species (*D. (I.) boreobaltica* sp.n. Salmela). Thus the range ("extent of occurrence") of *D. (I.) intricata* is actually smaller that was thought in the 2010 assessment. However, the species is not extremely rare and there are most likely hundreds of square kilometres of suitable breeding sites for the species in Finnish Lapland. Nevertheless, the species may be jeopardized by climate change and it may also be used as an indicator of pristine boreal mires.

#### Taxon discussion

See *Dicranomyia (I.) boreobaltica* Salmela sp.n.

#### DNA barcode

Standard 5′ region (658 bp) of the cytochrome *c* oxidase I (COI) gene of *Dicranomyia (I.) intricata* (BOLD Sample IDs JES-20120082 and JES-20110082, identical specimens):

TACCTTATACTTTATTTTTGGAGCTTGAGCAGGAATAGTAGGAACTTCACTAAGTATTATTATTCGAGCAGAATTAGGACACCCAGGAGCATTAATTGGAGATGACCAAATTTATAATGTAGTAGTTACTGCCCATGCTTTTATTATAATTTTTTTTATAGTTATACCAATTATAATTGGTGGATTCGGTAATTGATTAGTTCCTTTAATATTAGGAGCCCCAGATATAGCTTTCCCTCGAATAAATAATATAAGTTTTTGAATACTTCCCCCTTCTTTAACCTTATTATTAGCTAGAAGTATAGTTGAAAACGGGGCAGGAACTGGTTGAACAGTTTACCCTCCCCTTTCTTCTGGAATTGCTCATTCAGGAGCTTCTGTAGACTTAGCTATTTTTTCTCTTCATTTAGCAGGTATTTCTTCTATTTTAGGAGCTGTTAACTTTATTACAACTGTTATTAATATACGTTCAGCAGGAATTTCATTCGACCGAATACCATTATTTGTTTGATCAGTAGTAATTACTGCTATTCTATTACTCTTATCACTCCCTGTTTTAGCTGGAGCTATTACAATATTATTAACAGATCGAAACTTAAACACTTCATTTTTTGACCCTGCAGGTGGAGGAGATCCTATTTTATACCAACACTTATTT

## Analysis

The phylogenetic tree (length 622 steps) resulting from the parsimony analysis is shown in Fig. 13a and the optimal maximum likelihood tree (ln*L* = -3501.956818) in Fig. 13b. The results are not methodology-dependent since both parsimony and likelihood approaches resolve *D. (I.) boreobaltica* Salmela sp.n. as sister to *D. (I.) intricata* and these two species grouping together with *D. (I.) esbeni*. Nodal supports in both results are good giving strong indications that *D. (I.) boreobaltica* Salmela sp.n. forms its own group being in a sister group relationship with *D. (I.) intricata*. There are altogether 31 bases in COI that differ between *D. (I.) boreobaltica* Salmela sp.n. and *D. (I.) intricata*, resulting in 4.7 % difference between these species. Although nodal supports in the deeper, evolutionary older nodes are low, separate species are clearly distinct.

## Supplementary Material

Supplementary material 1 Dicranomyia (I.) intricata Alexander, 1927 (Diptera, Limoniidae), USNM Data type: images
Brief description: Non-type material of *Dicranomyia (I.) intricata* Alexander, males, permanently slide-mounted by C.P. Alexander, deposited in USNM (USA, Washington). Digital photos of the slides.
File: oo_33643.pdf Jukka Salmela

Supplementary material 2 COI sequences of Dicranomyia (Idiopyga) species and three out-group species Data type: Genomic
Brief description: COI 5' standard DNA barcoding fragment
File: oo_33852.txt Jukka Salmela

XML Treatment for Dicranomyia (Idiopyga) boreobaltica

XML Treatment for Dicranomyia (Idiopyga) intricata

## Figures and Tables

**Figure 2a.**
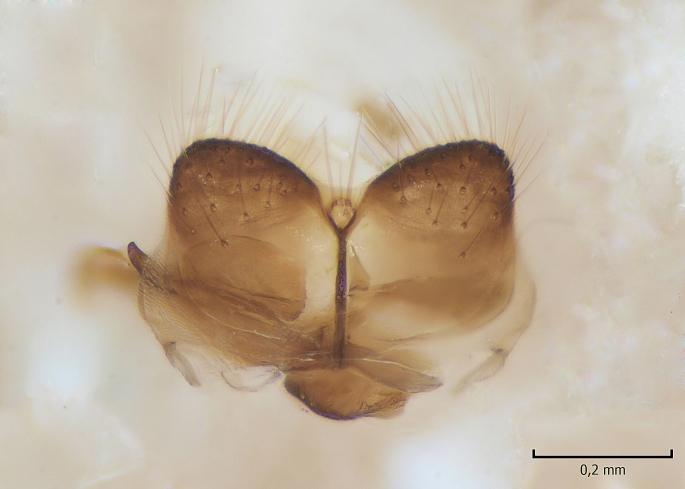
9th tergite, dorsal view.

**Figure 2b.**
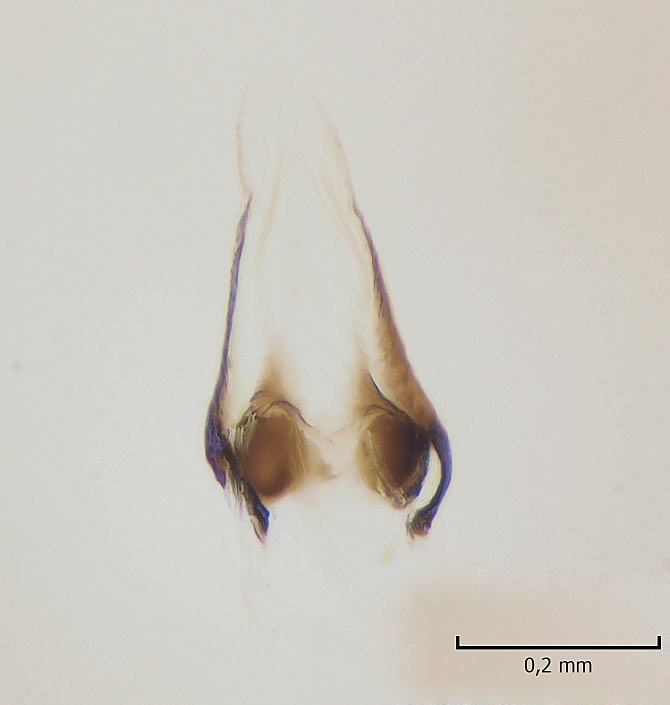
Proctiger, dorsal view.

**Figure 3a.**
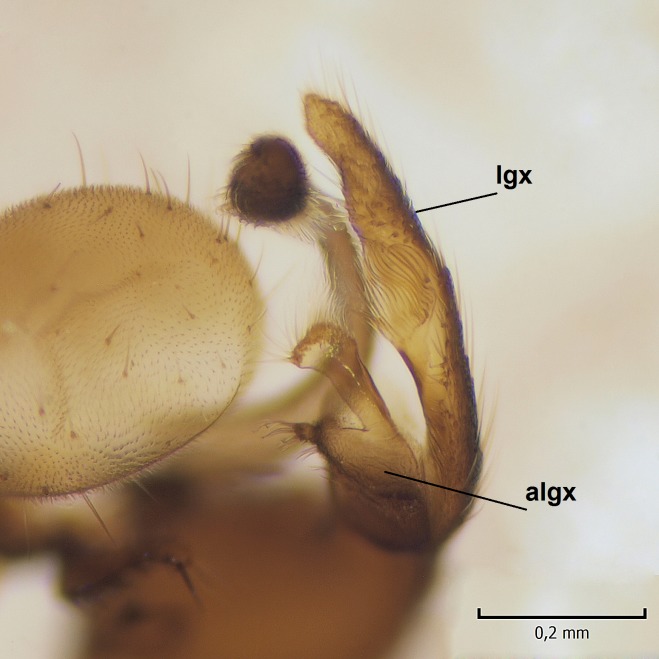
Gonocoxite and gonostylus, inner view.

**Figure 3b.**
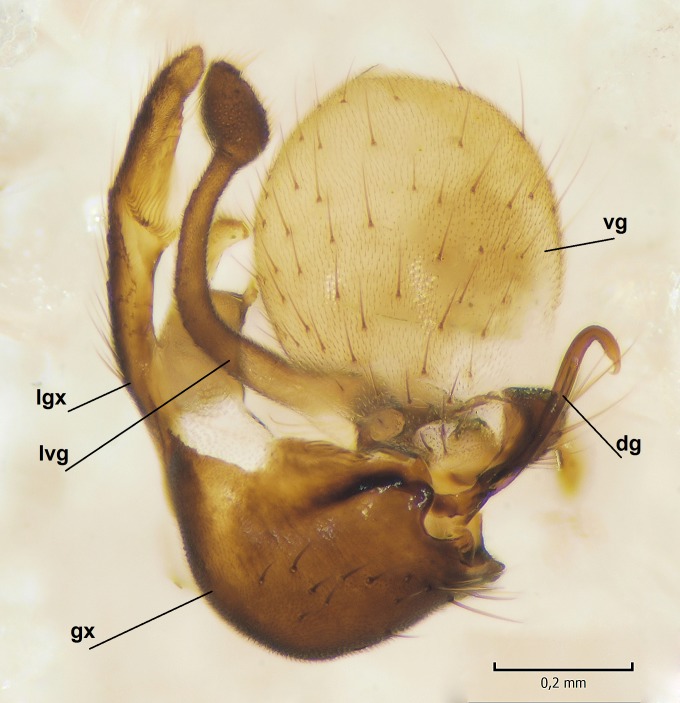
Gonocoxite and gonostylus, outer/lateral view.

**Figure 4a.**
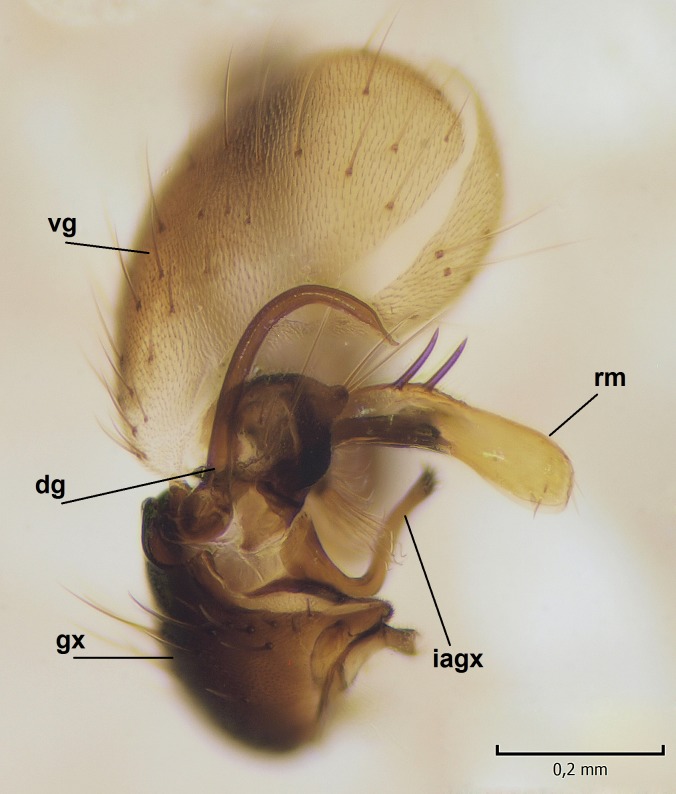
Gonostylus and gonocoxite, dorsal view.

**Figure 4b.**
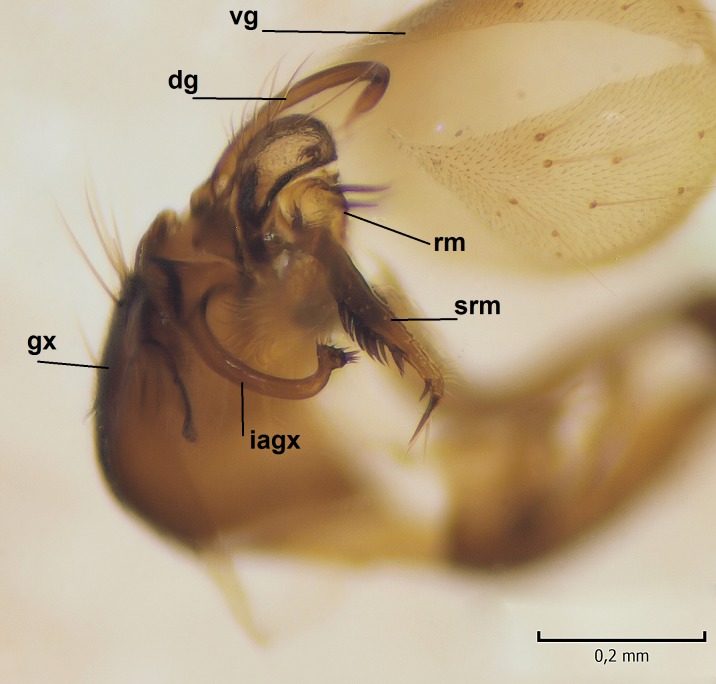
Gonostylus and gonocoxite, mesal view.

**Figure 4c.**
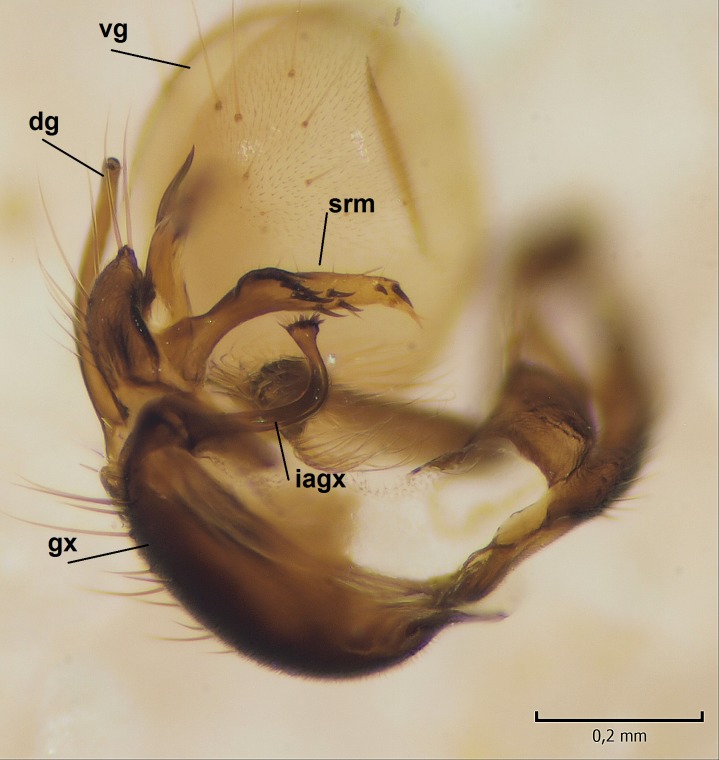
Gonostylus and gonocoxite, lateromesal view.

**Figure 4d.**
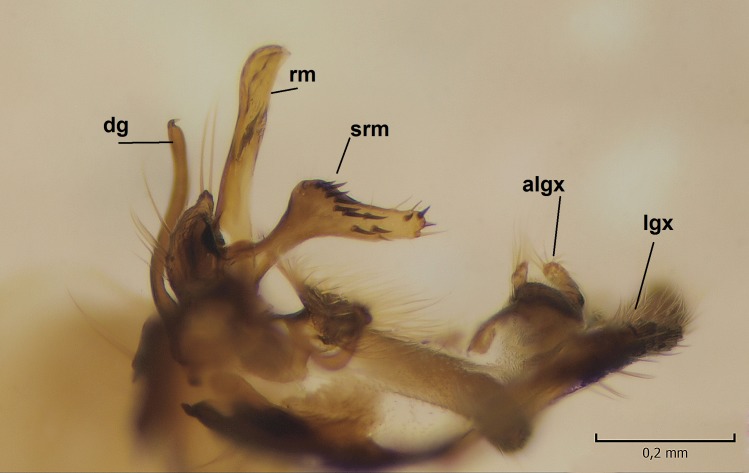
Gonostylus and gonocoxite, ventromesal view.

**Figure 5a.**
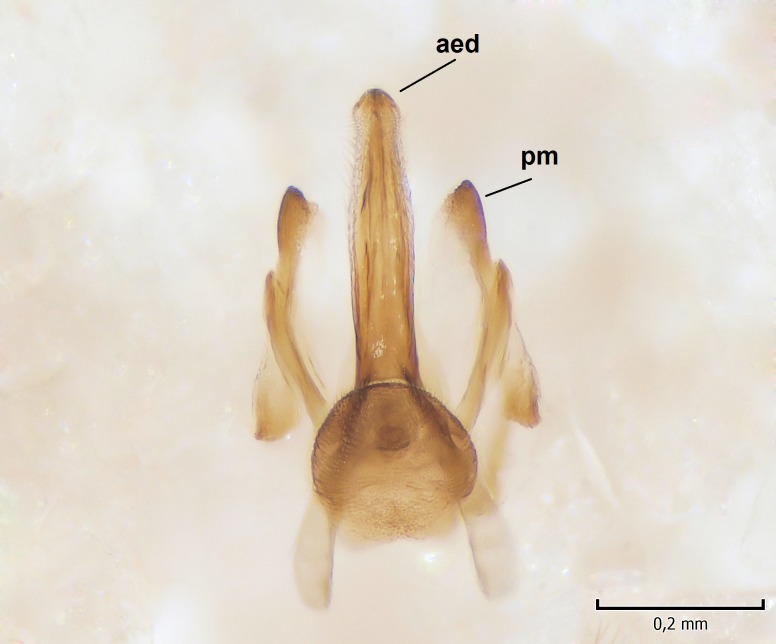
Ventral view.

**Figure 5b.**
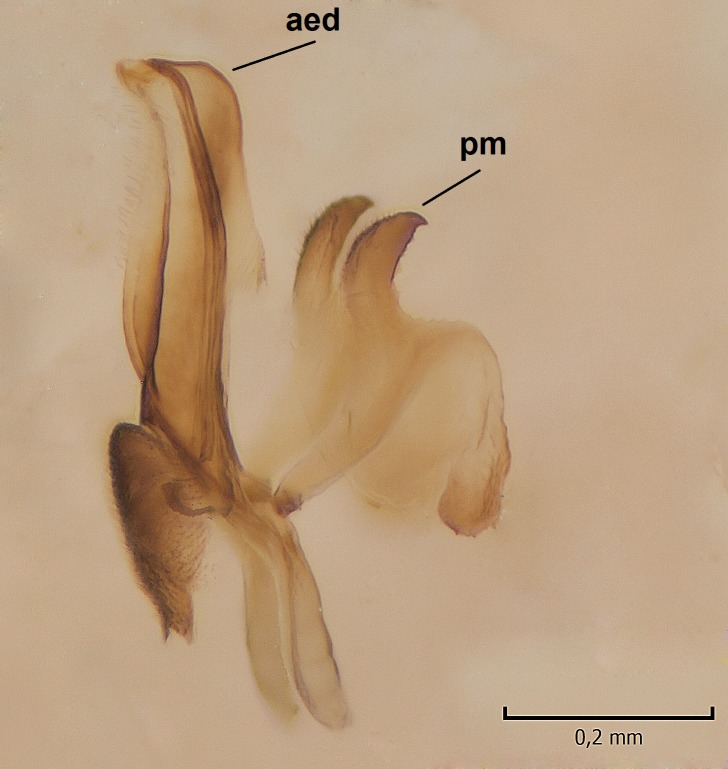
Lateral view.

**Figure 6a.**
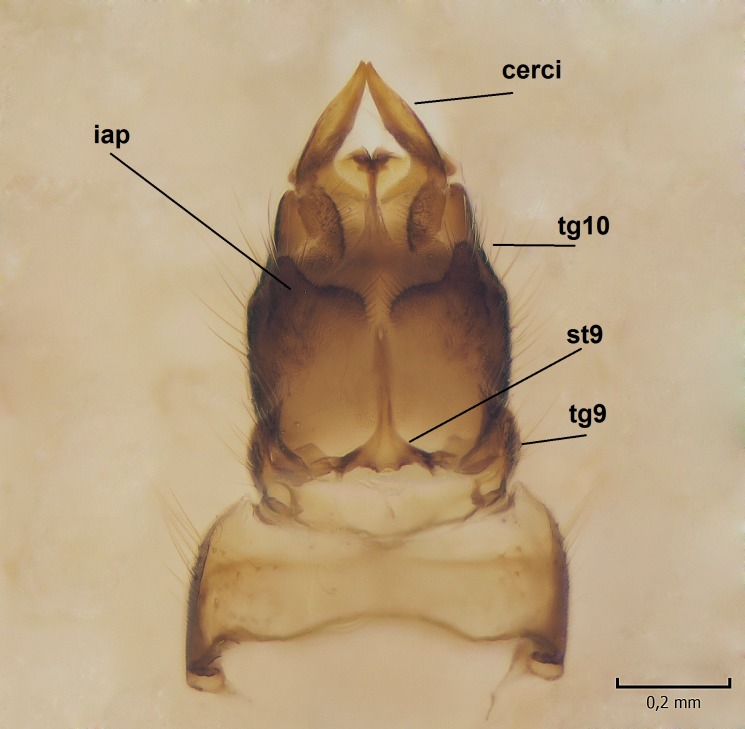
Cerci and 9th sternite, inner/ventral view. tg=tergite, st=sternite, iap=infra-anal plate. Vaginal apodeme is a triangular, hyaline membrane behind st9, which is not clearly seen in the photo.

**Figure 6b.**
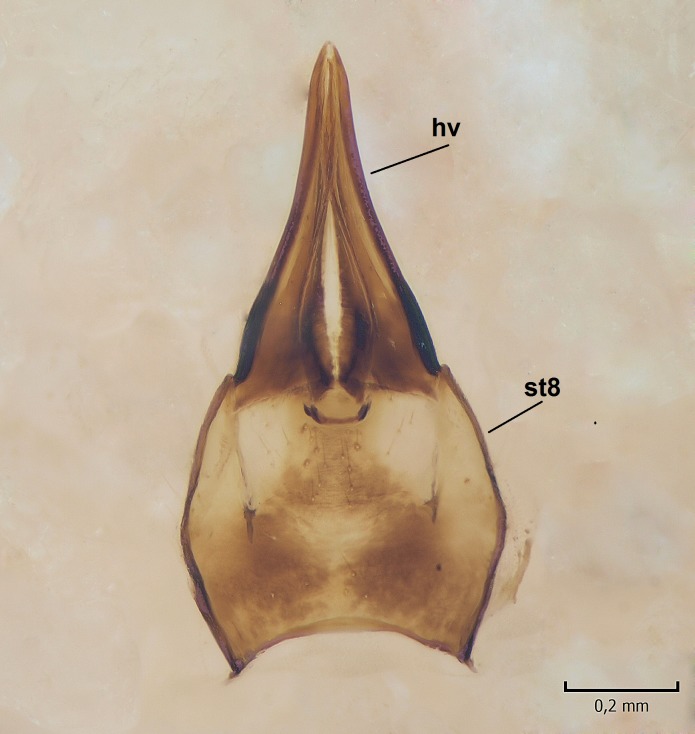
Hypogynial valves, inner/dorsal view. hv=hypogynial valves, st=sternite.

**Figure 7a.**
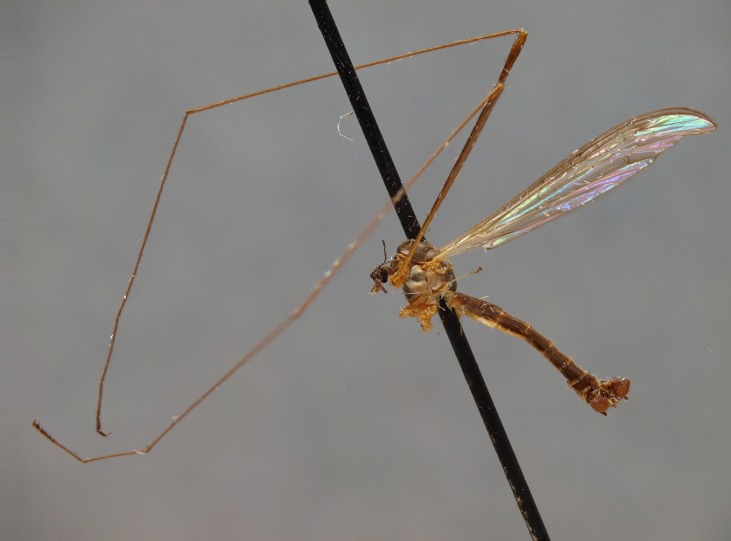
Holotype, male, habitus, lateral view.

**Figure 7b.**
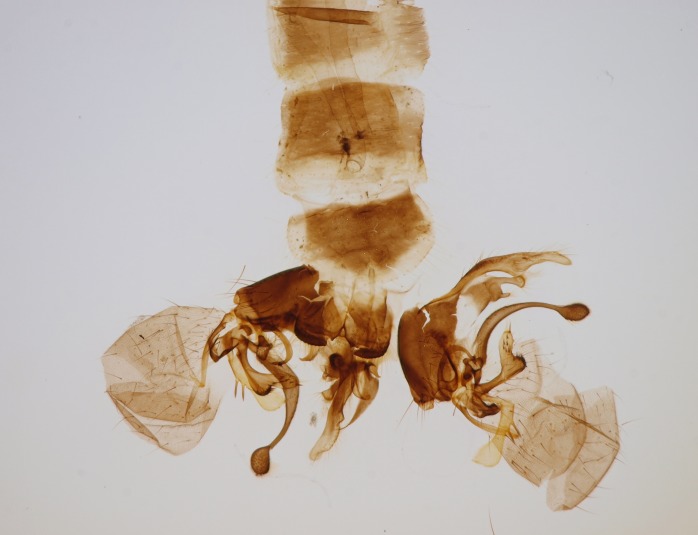
Paratype, male, abdominal terminalia and hypopygium, dorsal view. Permanent slide, perhaps mounted in Canada balsam.

**Figure 8a.**
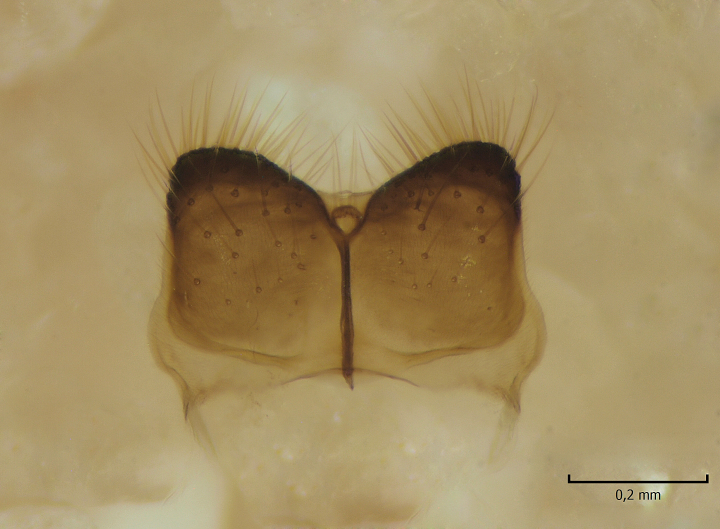
9th tergite, dorsal view.

**Figure 8b.**
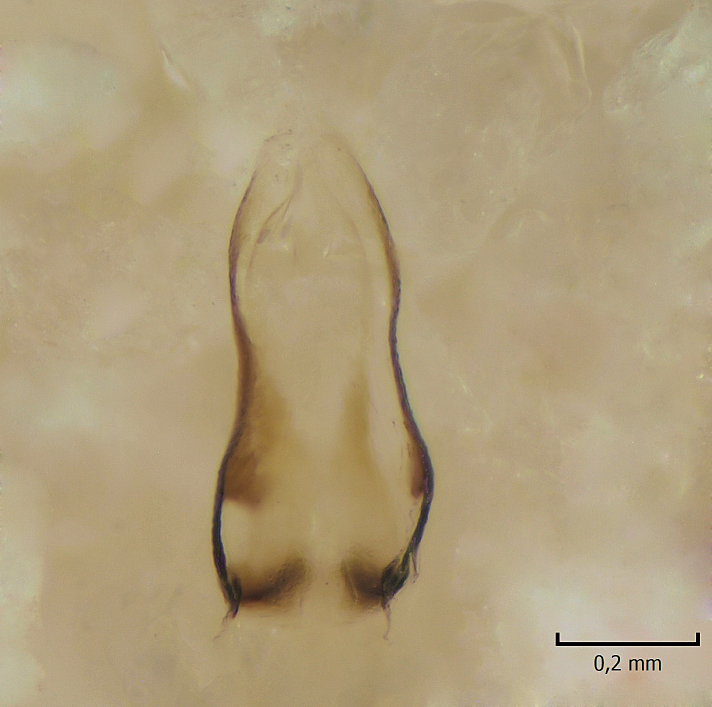
Proctiger, dorsal view.

**Figure 9a.**
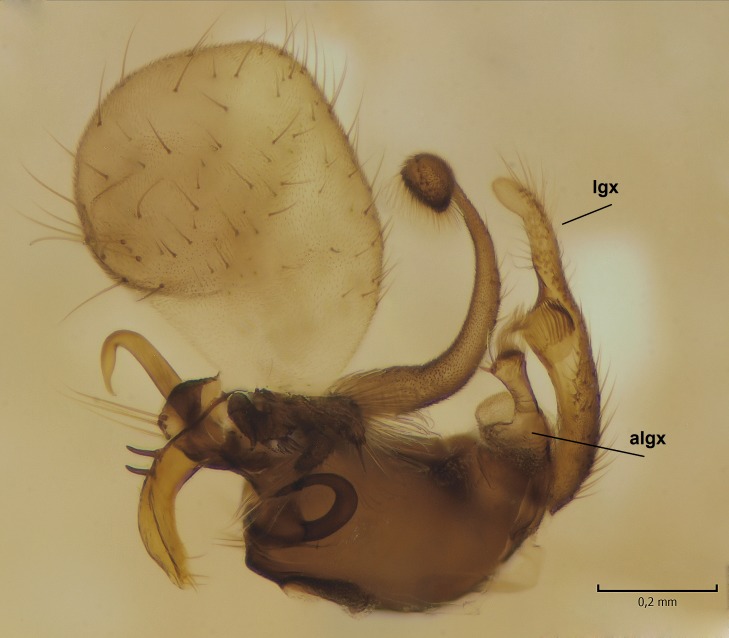
Gonocoxite and gonostylus, inner view.

**Figure 9b.**
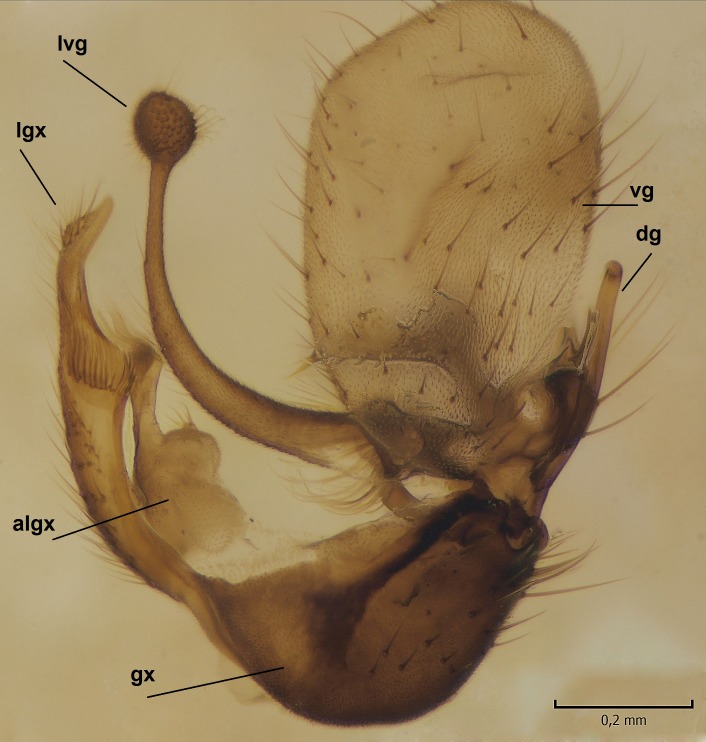
Gonocoxite and gonostylus, outer/lateral view.

**Figure 10a.**
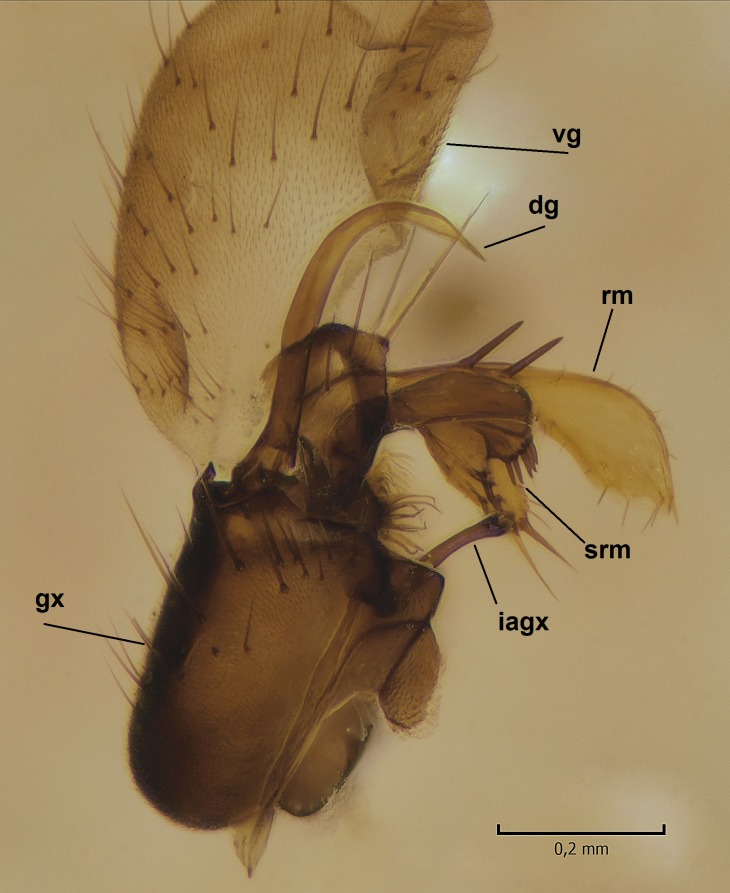
Gonocoxite and gonostylus, dorsal view.

**Figure 10b.**
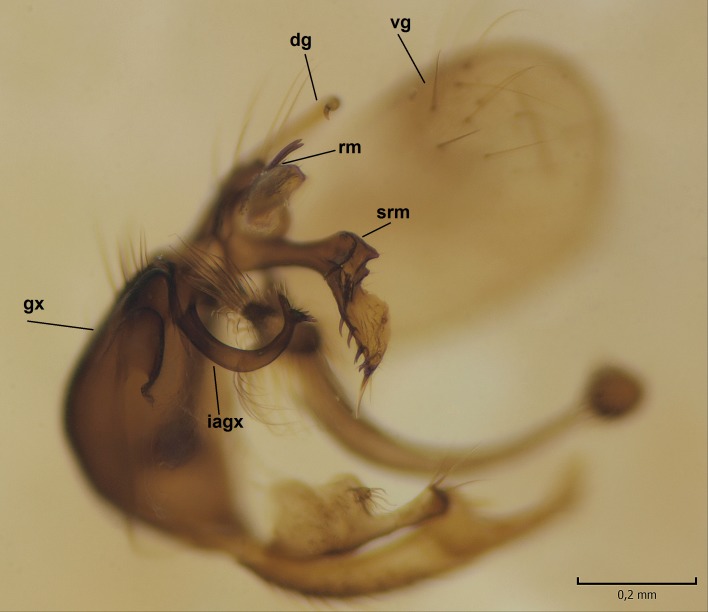
Gonocoxite and gonostylus, mesal view.

**Figure 10c.**
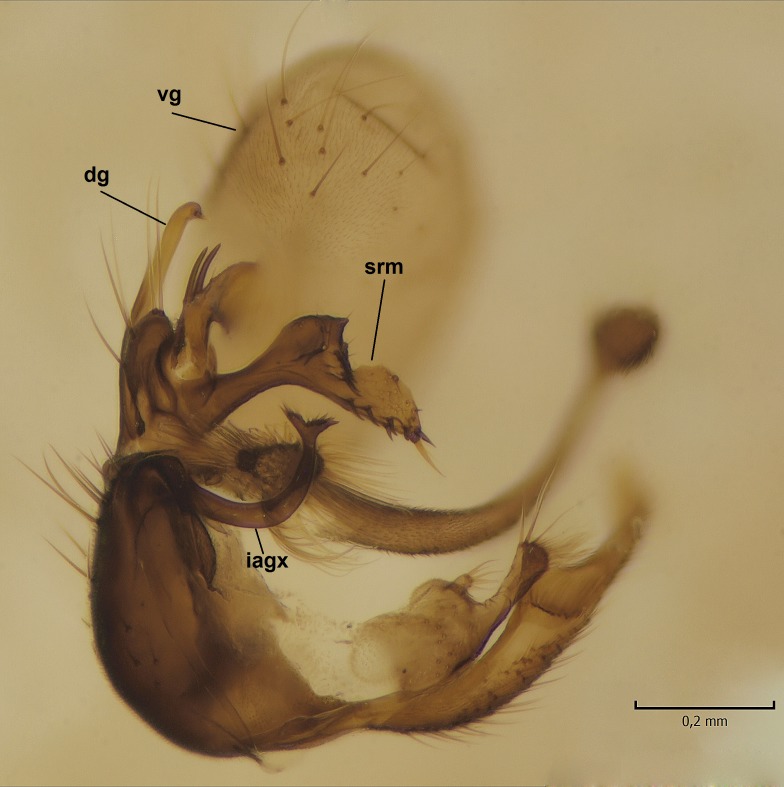
Gonostylus and gonocoxite, lateromesal view.

**Figure 10d.**
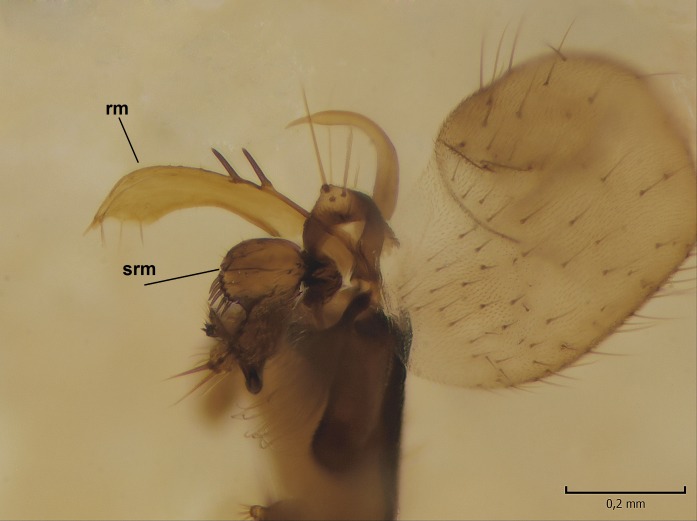
Gonostylus and gonocoxite, dorsomesal view.

**Figure 11a.**
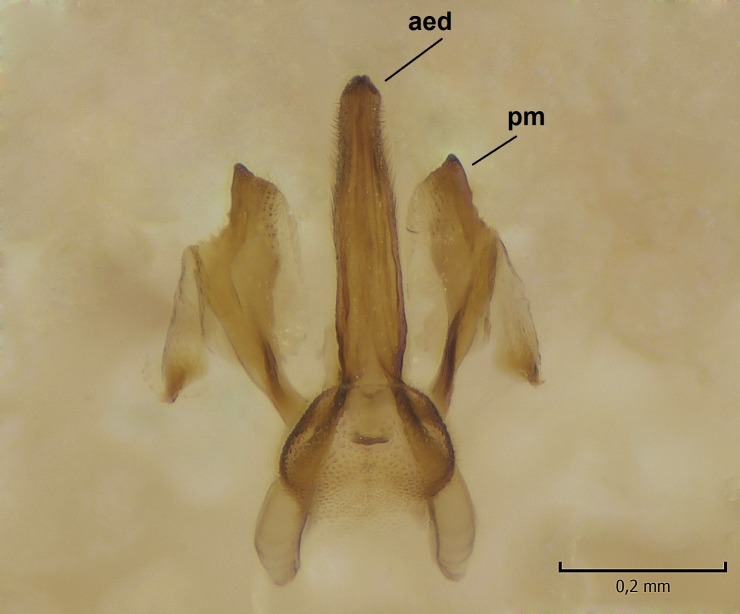
Ventral view.

**Figure 11b.**
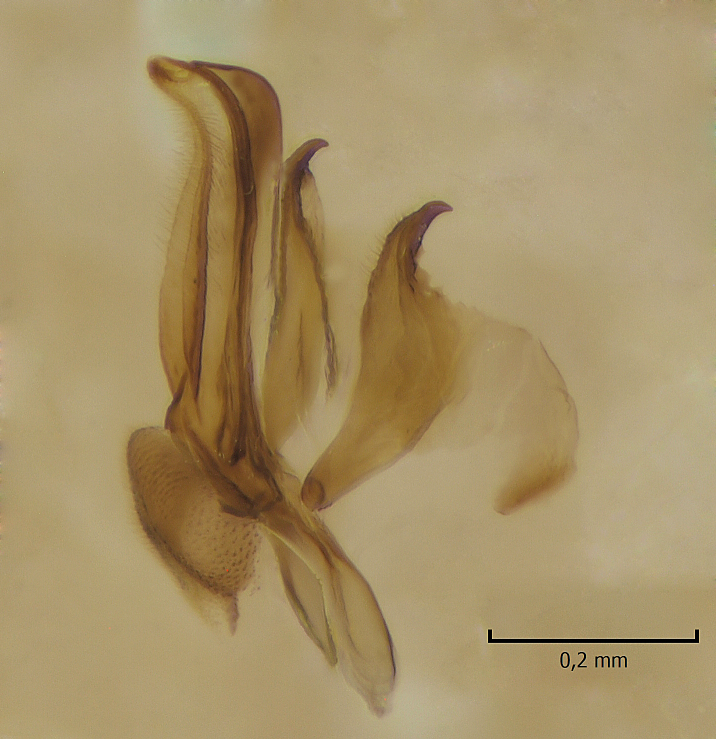
Lateral view.

**Figure 12a.**
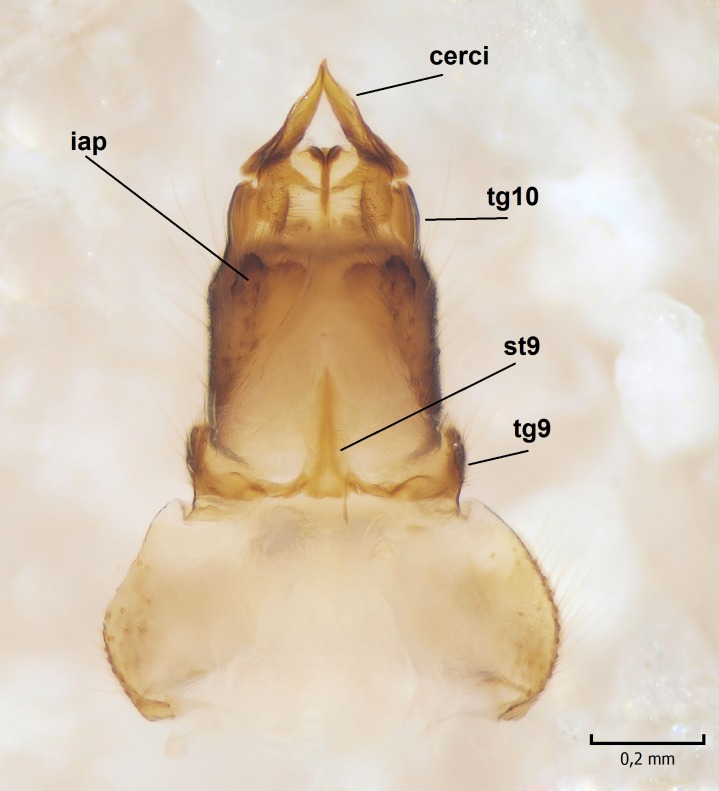
Cerci and 9th sternite, inner/ventral view. tg=tergite, st=sternite, iap=infra-anal plate. Vaginal apodeme (genital fork) is a triangular, hyaline membrane behind st9, not well visible in the photo.

**Figure 12b.**
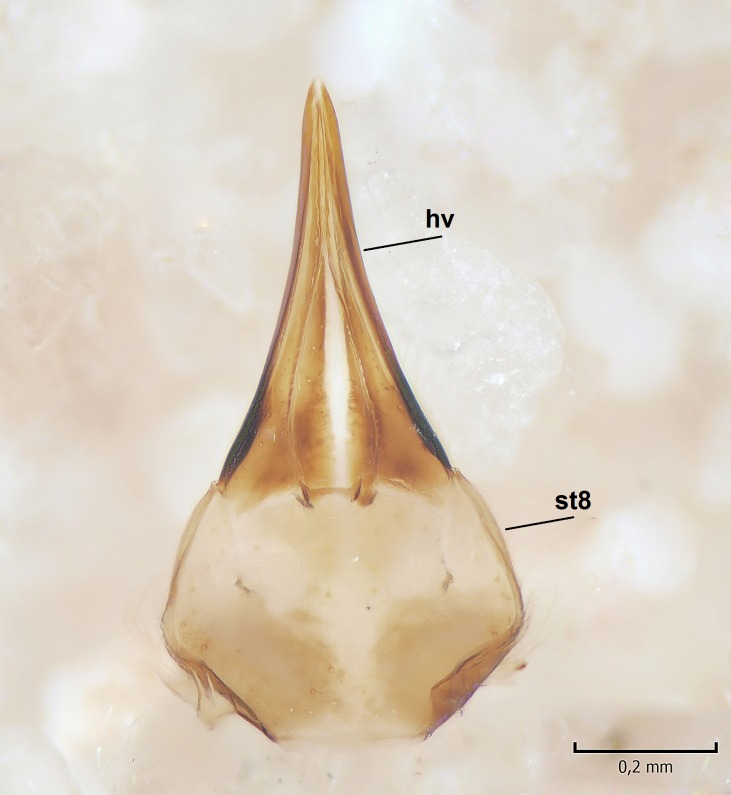
Hypogynial valves, inner/dorsal view. hv=hypogynial valves, st=sternite.

**Figure 13a.**
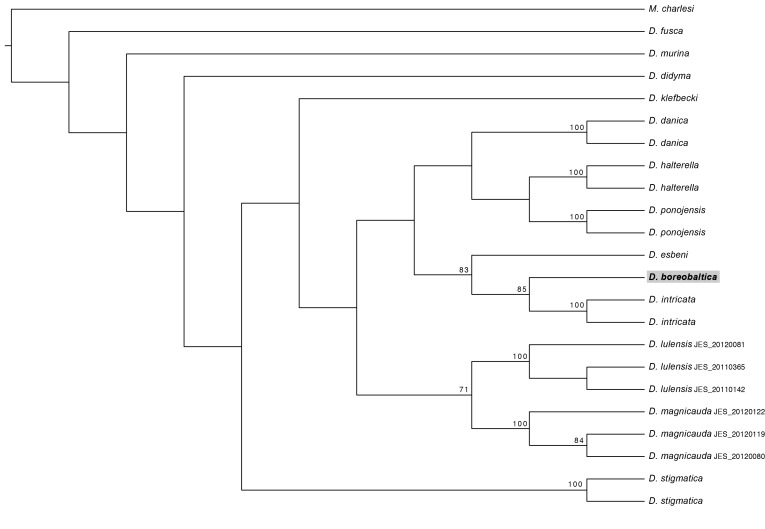
The single most parsimonius tree (L=622) with jackknife support on nodes.

**Figure 13b.**
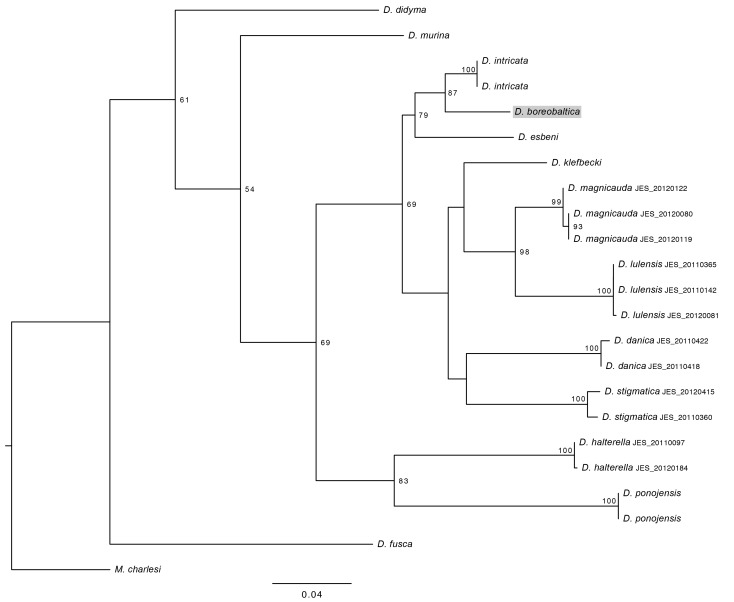
The optimal maximum likelihood tree (lnL= -3501.956818) with bootstrap support on nodes.

**Table 1.** *Dicranomyia (Idiopyga)* and outgroup specimens (*Dicranomyia (D.) didyma* (Meigen), *D. (Numantia) fusca* (Meigen), *Metalimnobia (M.) charlesi* Salmela & Starý) used in DNA barcoding (COI). Co-ordinates are given in WGS84 decimal format.

**species_sample ID**	**GenBank**	**year**	**country**	**locality**	**N**	**E**
Dicranomyia danica_JES-20110422	KP064166	2009	Czech Republic	Hrabetice	48.788	16.426
Dicranomyia danica_JES-20110418	KP064167	2009	Czech Republic	Hrabetice	48.788	16.426
Dicranomyia esbeni_JES-20120182	KP064169	2005	Finland	Oulunsalo	65.039	24.818
Dicranomyia halterella_JES-20120184	KP064171	2008	Finland	Kankaanpää	61.768	22.639
Dicranomyia halterella_JES-20110097	KP064172	2009	Finland	Enontekiö	68.636	22.784
Dicranomyia intricata_JES-20120082	KP064173	2009	Finland	Kittilä	67.639	25.427
Dicranomyia intricata_JES-20110082	KP064174	2009	Finland	Enontekiö	68.636	22.538
Dicranomyia boreobaltica_JES-20120094	KP064175	2005	Finland	Oulunsalo	64.906	25.376
Dicranomyia klefbecki_JES-20120377	KP064176	2011	Finland	Eckerö	60.253	19.541
Dicranomyia lulensis_JES-20120081	KP064177	2009	Finland	Kemijärvi	66.997	27.150
Dicranomyia lulensis_JES-20110365	KP064178	2007	Finland	Enontekiö	68.484	22.353
Dicranomyia lulensis_JES-20110142	KP064179	2009	Finland	Enontekiö	68.660	22.638
Dicranomyia magnicauda_JES-20120119	KP064180	2007	Finland	Kittilä	68.026	25.111
Dicranomyia magnicauda_JES-20120080	KP064181	2009	Finland	Kemijärvi	66.997	27.150
Dicranomyia magnicauda_JES-20120122	KP064182	2007	Finland	Kittilä	67.589	25.662
Dicranomyia murina_JES-20120042	KP064183	2009	Finland	Sodankylä	68.087	26.109
Dicranomyia ponojensis_JES-20110117	KP064184	2009	Finland	Enontekiö	68.636	22.538
Dicranomyia ponojensis_JES-20120086	KP064185	2007	Finland	Suomussalmi	65.230	28.170
Dicranomyia stigmatica_JES-20120415	KP064186	2006	Finland	Ruovesi	61.837	24.064
Dicranomyia stigmatica_JES-20110360	KP064187	2007	Finland	Kittilä	67.593	25.308
Dicranomyia didyma_JES-20110098	KP064168	2009	Finland	Enontekiö	68.636	22.784
Dicranomyia fusca_JES-20110237	KP064170	2008	Finland	Nurmes	63.786	29.350
Metalimnobia charlesi_JES-20110381	KP064165	2008	Finland	Lieksa	63.468	29.942

**Table 2.** Summary of the most important postabdominal differences between *Dicranomyia (I.) boreobaltica* Salmela sp.n. and *D. (I.) intricata* Alexander.

***D. (I.) boreobaltica***	***D. (I.) intricata***
apex of iagx simple, not furcated (Fig. 4c)	apex of iagx bifurcated (Fig. 10b, c)
apex of lgx angular (Fig. 3)	apex of lxg beak-like (Fig. 9)
stalk of lvg rather wide, apex oval (Fig. 3)	stalk of lvg tapering apically, apex spherical (Fig. 9)
apex of rm rounded, rather narrow (Fig. 4a)	apex of rm pointed, rather wide (Fig. 10a)
srm simple, not bilobed (Fig. 4b, c, d)	srm bilobed (Fig. 10b, c, d)
caudal margin of female infra-anal plate as in Fig. 6a	caudal margin of female infra-anal plate as in Fig. 12a
