# Supplementary material for: Unveiling of a cryptic Dicranomyia (Idiopyga) from northern Finland using integrative approach (Diptera, Limoniidae)
Source: Biodivers Data J. 2014 Dec 3;(2):e4238. doi: 10.3897/BDJ.2.e4238 (PMC4267105; doi:10.3897/BDJ.2.e4238)
Supplement: Supplementary material 1 — Dicranomyia (I.) intricata Alexander, 1927 (Diptera, Limoniidae), USNM [file biodiversity_data_journal-2-e4238-s001.pdf]

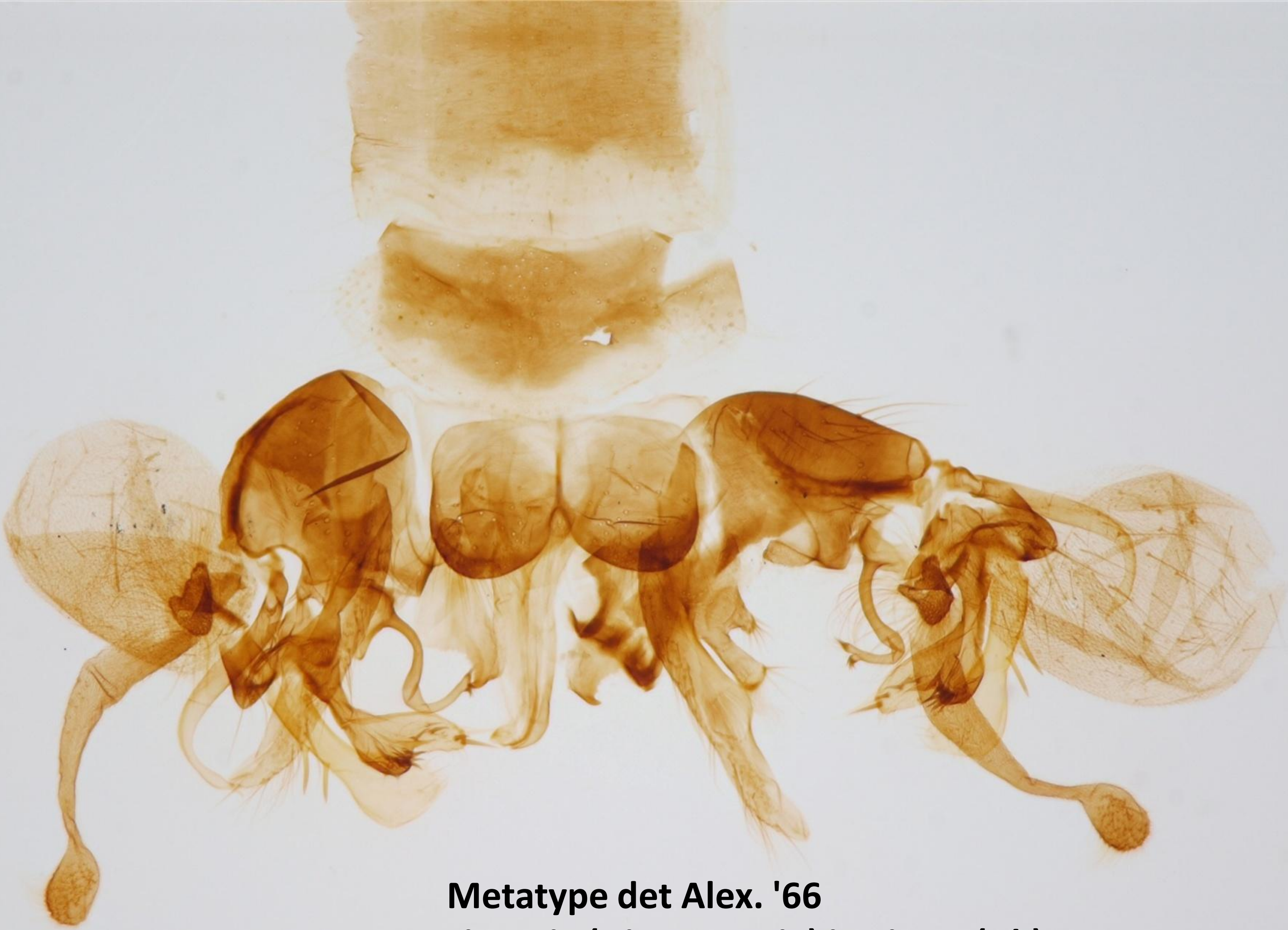

Metatype det Alex. '66
Limonia (Dicranomyia) intricata (Al.)
Yukon
Dawson
1100'
Aug.6, 1949 P.T. Bruggemann (3)

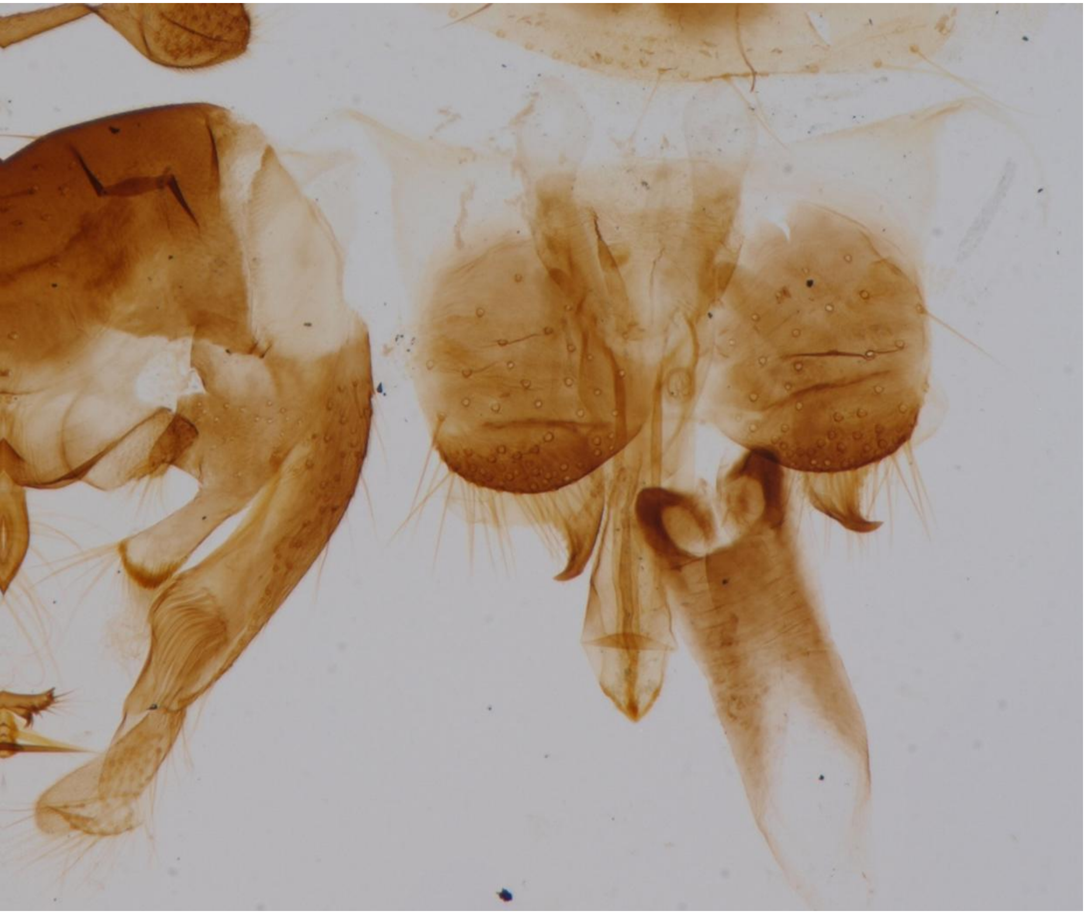

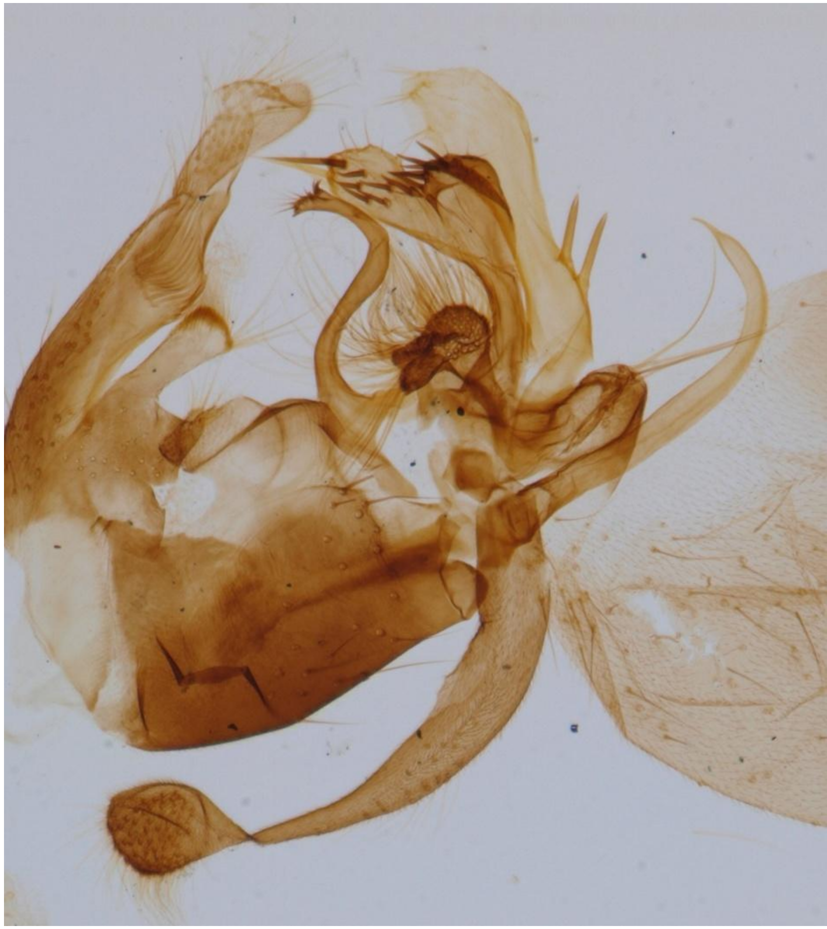

Limonia (Dicranomyia) intricata (Al)
Brit. Columbia
Telegraph Creek
1100' Aug. 28 '60 W.W. Moss
The Alexander Collection of Crane flies 3586
Met 4

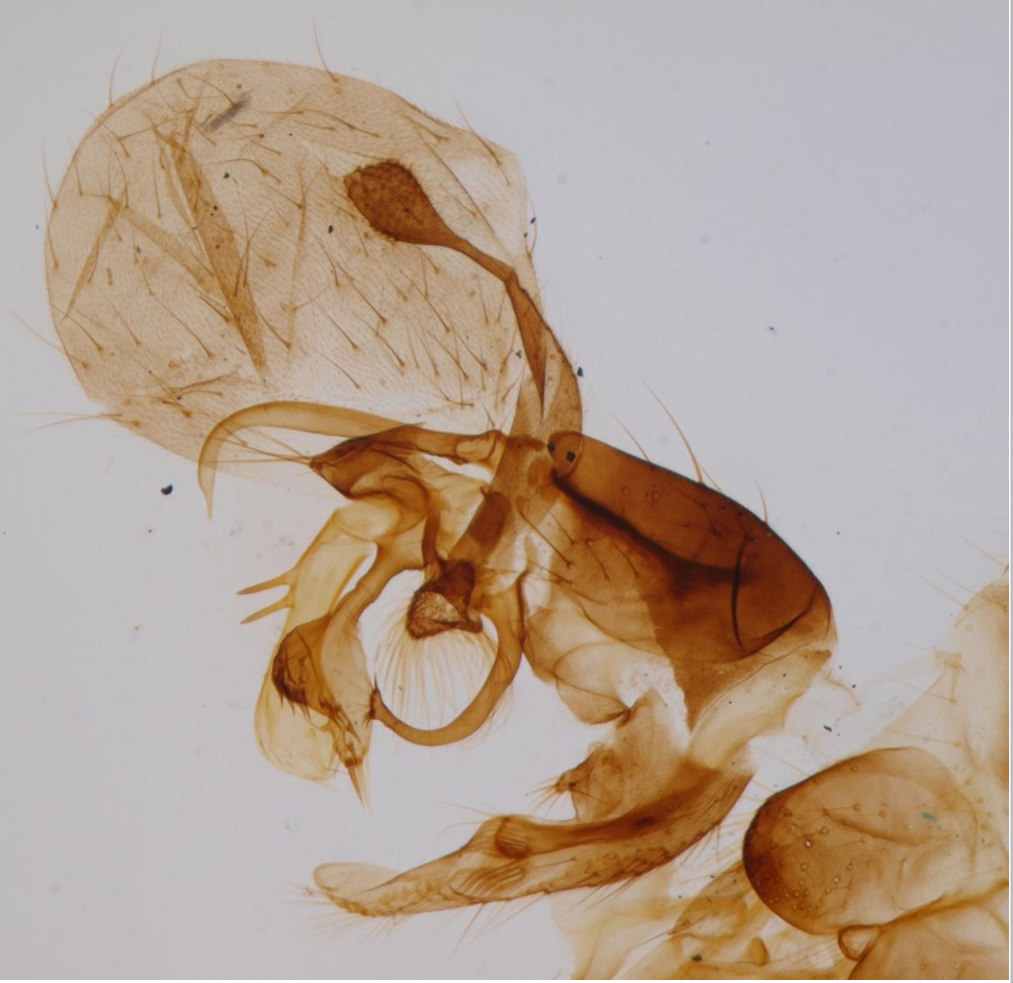

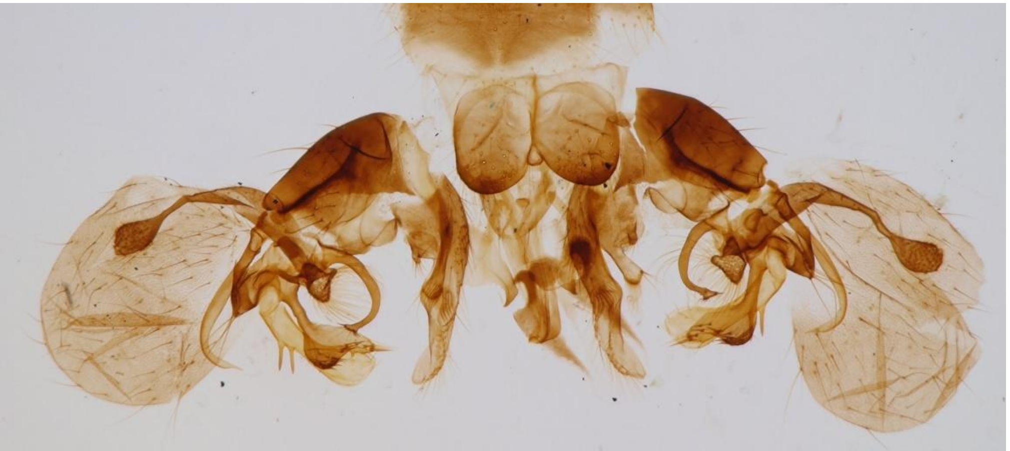

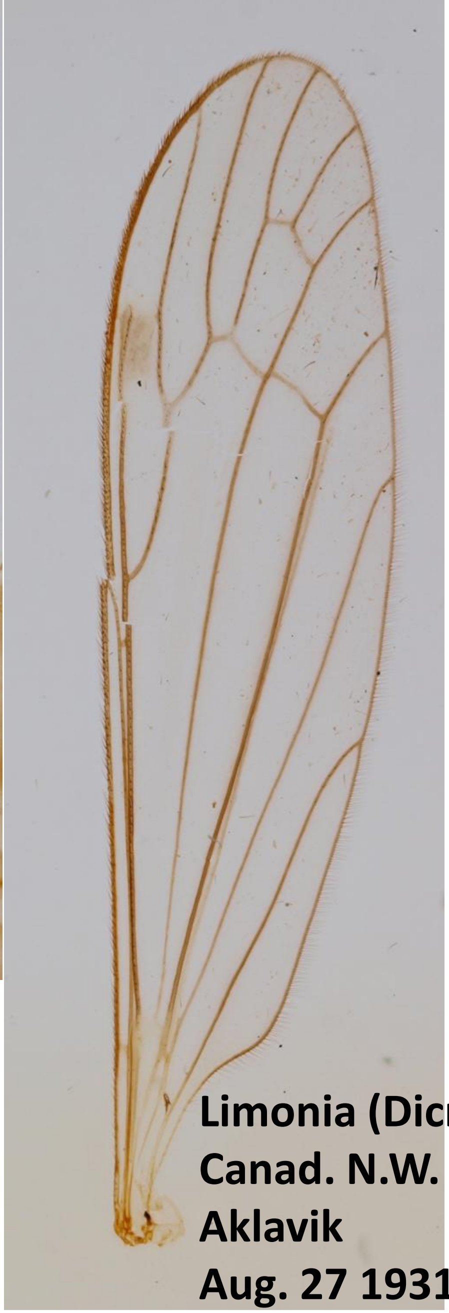

Limonia (Dicranomyia) intricata (Al)
Canad. N.W. Terr
Aklavik
Aug. 27 1931
O. Bryant 257
Met. 3586

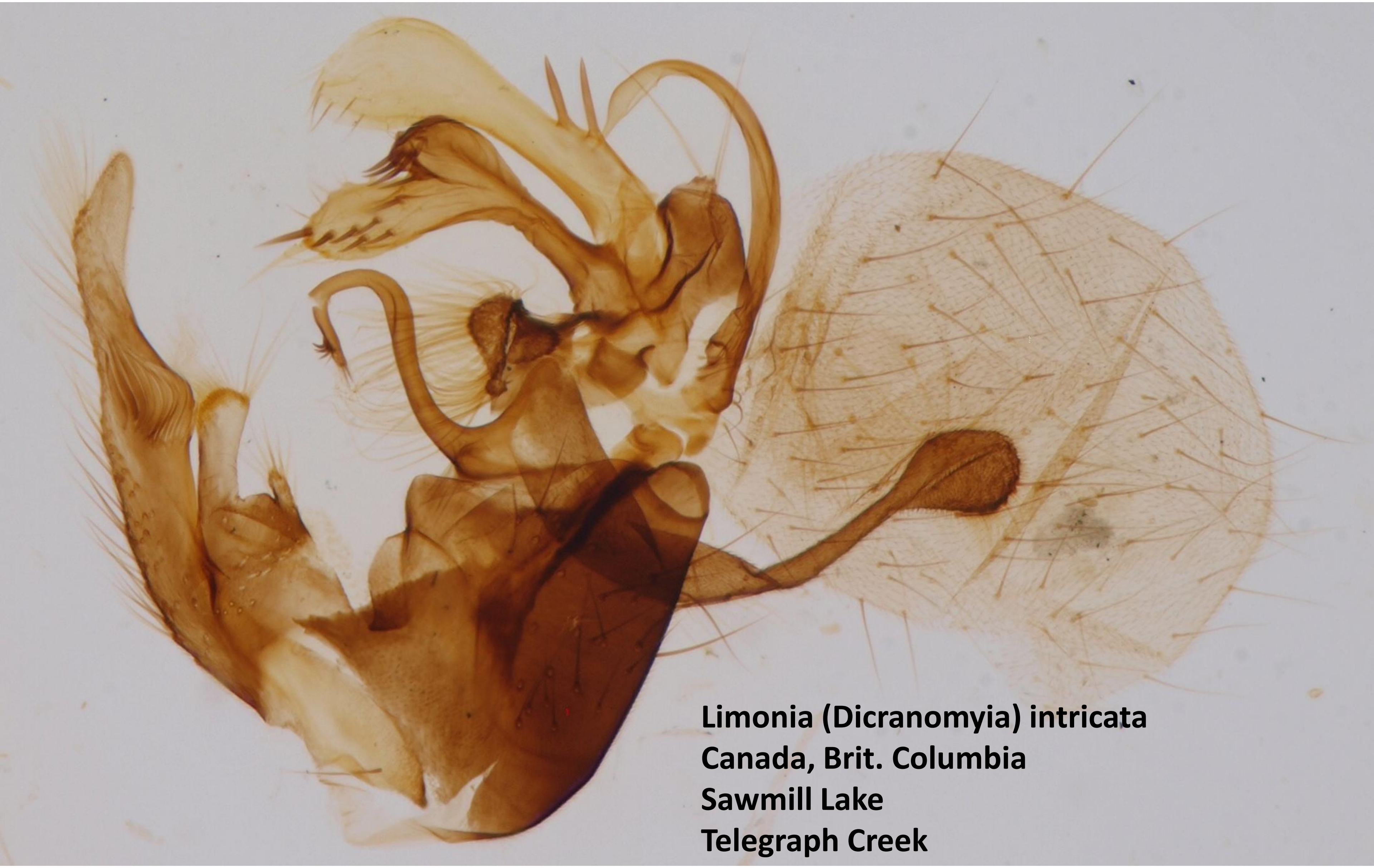

Limonia (Dicranomyia) intricata
Canada, Brit. Columbia
Sawmill Lake
Telegraph Creek
VIII 18 1960
W.W. Moss
Met 3586
